# Development of transgenic *Brassica juncea* lines for reduced seed sinapine content by perturbing phenylpropanoid pathway genes

**DOI:** 10.1371/journal.pone.0182747

**Published:** 2017-08-07

**Authors:** Sachin Kajla, Arundhati Mukhopadhyay, Akshay K. Pradhan

**Affiliations:** 1 Department of Genetics, University of Delhi South Campus, New Delhi, India; 2 Centre for Genetic Manipulation of Crop Plants, University of Delhi South Campus, New Delhi, India; New South Wales Department of Primary Industries, AUSTRALIA

## Abstract

Sinapine is a major anti-nutritive compound that accumulates in the seeds of *Brassica* species. When ingested, sinapine imparts gritty flavuor in meat and milk of animals and fishy odor to eggs of brown egg layers, thereby compromising the potential use of the valuable protein rich seed meal. Sinapine content in *Brassica juncea* germplasm ranges from 6.7 to 15.1 mg/g of dry seed weight (DSW) which is significantly higher than the prescribed permissible level of 3.0 mg/g of DSW. Due to limited natural genetic variability, conventional plant breeding approach for reducing the sinapine content has largely been unsuccessful. Hence, transgenic approach for gene silencing was adopted by targeting two genes—*SGT* and *SCT*, encoding enzymes UDP- glucose: sinapate glucosyltransferase and sinapoylglucose: choline sinapoyltransferase, respectively, involved in the final two steps of sinapine biosynthetic pathway. These two genes were isolated from *B*. *juncea* and eight silencing constructs were developed using three different RNA silencing approaches viz. antisense RNA, RNAi and artificial microRNA. Transgenics in *B*. *juncea* were developed following *Agrobacterium*-mediated transformation. From a total of 1232 independent T_0_ transgenic events obtained using eight silencing constructs, 25 homozygous lines showing single gene inheritance were identified in the T_2_ generation. Reduction of seed sinapine content in these lines ranged from 15.8% to 67.2%; the line with maximum reduction had sinapine content of 3.79 mg/g of DSW. The study also revealed that RNAi method was more efficient than the other two methods used in this study.

## Introduction

Seeds of oilseed *Brassica* species (rapeseed-mustard) contain oil at 40–50% of the dry dry seed weight (DSW) and are also rich in valuable proteins. The proteins have well balanced amino acid composition with protein efficiency ratio comparing favourably with that of milk and beef [1] and like soy proteins has the potential to be used as food supplement. However, the presence of certain undesirable components, especially phenolic compounds, has foiled the use of the seed meal as food supplement [2].

Sinapate esters are considered as the most prevailing phenolic compounds in brassica seeds with sinapoylcholine (sinapine) as the major component [2]. It is synthesized exclusively during seed filling stages and accumulates in the embryo [3]. During oil extraction sinapate esters get oxidized and form complexes with proteins, thus diminishing the digestibility of brassica seed meal [2,4,5]. Sinapine bestows a gritty flavor in milk and meat of animals that consume diet enriched with brassica seed meal and confer fishy odor in brown-shelled eggs [2,5–6]. When consumed in excess, it causes serious growth and reproductive problems in animals [1,6–9].

Sinapine is synthesized from phenylalanine that is converted to sinapate through a series of methylation and hydroxylation steps via phenylpropanoid pathway. During seed filling stage sinapate is catalyzed by UDP- glucose: sinapate glucosyltransferase (SGT) enzyme and is converted to 1-*O*- sinapoylglucose, which in turn is acted upon by sinapoylglucose: choline sinapoyltransferase (SCT) enzyme to produce sinapine [10–11].

Rapeseed-mustard is cultivated worldwide for its oil. *B*. *juncea* is the second most important oil seed crop in India contributing nearly 25–30% of the total oil seed production in the country [12]. *B*. *juncea* (AABB) is an allopolyploid species containing the genomes of two diploid species namely, *B*. *rapa* (AA) and *B*. *nigra* (BB). Sinapine content in *B*. *juncea* has been reported to be 13 mg/g of DSW [13]. The acceptable quantity of sinapate esters for consumption by animals has been estimated to be 2–3 mg/g of DSW and for humans it is below 1 mg/g DSW [2]. Our initial estimation of 76 *B*. *juncea* lines revealed that sinapine content in the seeds of *B*. *juncea* ranges from 6.7 to 15.13 mg/g DSW. It indicated that there is no germplasm in *B*. *juncea* with sufficiently low seed sinapine content that can be used as a donor source for developing low sinapine mustard variety through conventional breeding. Attempt to develop low sinapine lines using chemical mutagens also did not yield desirable result [14]. Hence, a transgenic approach, aimed at disrupting the activity of the key enzymes involved in the biosynthetic pathway of sinapine was thought to be a better alternative for developing low sinapine lines in mustard.

In this study, we report the development of low sinapine mustard lines with significant reduction of seed sinapine content following the transgenic approach. Eight suppression constructs against two key genes (*SGT* and *SCT*), involved in catalyzing the two terminal steps in sinapine biosynthetic pathway, were developed and transgenic *B*. *juncea* obtained following *Agrobacterium tumefaciens* mediated transformation. Characterization and evaluation of a total of 1232 independent transgenics over three generations (T_0_ to T_2_) led to the establishment of 25 single-gene homozygous T_2_ transgenic lines showing up to 67.2% reduction of seed sinapine content compared to that of the wild type.

## Materials and methods

### Plant material

*B*. *juncea* cultivar Varuna was used for *Agrobacterium*-mediated genetic transformation.

### Isolation of genes and promoter sequences from *B*. *juncea*

Genomic DNA was isolated according to the protocol of Rogers and Bendich (1994) [15] from well expanded leaves of field grown plants. Total RNA was isolated from immature seeds using Spectrum Total RNA Isolation Kit (Sigma-Aldrich, St Louis, MO) following manufacturer’s instructions. Approximately, 2.0 μg of total RNA was reverse transcribed using High Capacity cDNA Reverse Transcription Kit (Applied Biosystem, Foster city, CA) according to manufacturers’ instructions.

The sequence information for the homologs of *SGT* and *SCT* genes is available for different *Brassica* species (*B*. *rapa*, *B*. *oleracea* and *B*. *napus*) and *A*. *thaliana* on EMBL (www.ebi.ac.uk) and TAIR (www.arabidopsis.org) databases. For *SGT* gene, *B*. *rapa* [two homologs (Accession numbers FM872282 and FM872283)], *B*. *oleracea* [two homologs (Accession numbers FM872280 and FM872281)], *B*. *napus* [four homologs (Accession numbers FM872276, FM872277, FM872278 and FM872279)] and *A*. *thaliana* (At3g21560) sequences were aligned and used for designing of a primer pair (BjSGT- IT-F and BjSGT- IT-R; **S1 Table**) for the amplification of full length gene from the genomic DNA of *B*. *juncea*.

For *SCT* gene, *B*. *rapa* [one homolog (Accession number AM706348)], *B*. *oleracea* [one homolog (Accession number AM706347)], *B*. *napus* [two homologs (Accession numbers AM706349 and AM706350)] and *A*. *thaliana* [one homolog (At5g09640)] sequences were aligned and used for designing a primer pair (BjSCT-F and *BjSCT*-R; **S1 Table**) for the amplification of full length coding sequences from the cDNA library of *B*. *juncea*.

The 5´upstream region of the *SCT* gene was isolated by genome walking using GenomeWalker universal kit (Clontech Lab. Inc., USA). Nested primers (**S1 Table**) were designed from 5´-end of the coding region using Six GenomeWalker libraries for the restriction enzymes namely, *Sca* I, *Dra* I, *EcoR* V, *Pvu* II, *Stu* I and *Ssp* I. PCR amplification was conducted following manufacturer’s instructions.

### *In-silico* structural analysis and molecular docking

The *in-silico* structural analysis and molecular docking were performed following Rajput et al. (2013) [16]. In brief, the predicted amino acid sequences of SGT and SCT enzymes were subjected to PSI-Blast search against PDB database to select the suitable template for homology modelling. Multifunctional (Iso) flavonoid Glycosyltransferase (PDB id: 2PQ6) [17] was selected as template for SGT and human ‘protective protein’ (HPP, PDB id: 1IVY) [18] for SCT enzyme. The Modeller 9.11 was used for homology modelling of both the enzymes. Side chain modifications were done by SCWRL and optimum bond length and bond angles were attained by energy minimization using AMBER force field inbuilt in GROMACS 4.5.4 package. Three-dimensional structural validation of modeled structures was performed by the structural analyses and verification server (SAVS server) using PROCHECK and WHATCHECK programs. Secondary structure and other structural parameters were analyzed by PSIPRED and VADAR, respectively. The superimpositions of C_α_ atoms of modeled structures with their respective templates and visualizations of proteins and structural alignments were performed via Pymol. Further, pocket finder and CASTp were used for identifications of the probable active sites. Molecular modeling of substrates sinapate and 1-*O*-sinapolglucose was performed by PRODRG. Patch dock tool was used for docking studies of both enzymes with their respective substrates. In each case, the top 20 docked complexes were retrieved and analyzed. The most suitable complex was selected in each case on the basis of best docking score and best-fitting ligand pose in the binding cavity.

### Development of suppression constructs for *SGT* and *SCT* genes

A total of eight different silencing constructs, four each for *SGT* and *SCT* genes viz. antisense RNA (BjSGTAS for *SGT* and BjSCTAS for *SCT*), RNAi (BjSGTRNAi for *SGT* and BjSCTRNAi for *SCT*), and artificial microRNA (BjSGTamiR38 and BjSGTamiR40 for *SGT*; BjSCTamiR36 and BjSCTamiR37 for *SCT*) were developed. (**S1 Fig**). Detailed procedure for developing different constructs is given in **S1 Appendix**. Four suppression constructs of *SGT* gene were driven by seed specific *napin* gene promoter [19] while four suppression constructs of *SCT* gene were driven by *SCT* gene endogenous promoter (750 bp upstream region of *SCT* gene). In all these constructs, polyA tail of octopine synthase gene was used as the terminator and *bar* gene, driven by 35S promoter with double enhancer was used as both *in vitro* and field selection marker for resistance against herbicide basta (**S1 Fig**).

### Development of transgenic lines

Transformation of *B*. *juncea* cv. Varuna was performed by *Agrobacterium-*mediated genetic transformation using hypocotyl explants following the protocol described by Mehra et al. (2000) [20]. Transgenic plants were grown in containment net house (as per the guidelines of Department of Biotechnology, Government of India) in the growing season (October–March) or in CONVIRON plant growth chambers, maintained at 25°C–18°C and 14h–10h day/night cycle at 80% RH. Screening for basta resistant plants was done either by coating (T_0_ generation) or spraying (T_1_ and T_2_ generations) the leaves with basta solution (200mg/l). Basta resistant plants were maintained through selfing.

#### Molecular characterization

Transgene detection: The detection of *bar* gene was done by PCR amplification of a 550 bp from the *bar* gene (Bj-bar-F and Bj-bar-R; **S1 Table**) in the T_0_ generation. The presence of transgenes was assessed in T_1_ generation through PCR amplification by designing insert specific primer pairs for different silencing constructs (**S2 Fig**).

Southern hybridization: Approximately 10 μg of genomic DNA was digested with *Eco* RI restriction endonuclease, resolved on 0.8% agarose gel and was transferred to nylon membrane (Amersham Hybond^+^, GE Healthcare, UK). Southern hybridization was performed following Sivaraman et al (2004) using a 500 bp fragment (probe) of *bar* gene as shown in **S3 Fig**.

Transcripts analysis using qRT-PCR: Relative expression analyses of genes were performed using Eppendorf Realplex (Eppendorf, Hamburg, Germany), real time PCR machine. Primers were designed from the conserved regions of the genes so that it can amplify all the functional paralogs. SYBR green method was used for the assessment of transcripts. Ubiquitin gene was used as the internal control; data were analysed in three independent biological replicates with three technical replicates each. Statistical analysis was performed using one-way ANOVA followed by post hoc LSD test of significance, wherever necessary. The gene specific primers that were designed are listed in **S1 Table**.

### Sinapine extraction and estimation

Extraction and estimation of seed sinapine content was done following Hüsken et al. (2005) [2]. Seeds were ground to a fine powder using mortar and pestle. Nine hundred microliter of extraction solvent (80% methanol containing 1.5% acetic acid) was added to 60 mg of powdered seed and vortexed for 3 min. The samples were then incubated at 30°C for 15 min in a water bath and then vortexed briefly. This process was repeated once and samples were then stored at -20°C for half an hour. The samples were centrifuged at 13000 rpm for 15 min and the supernatants were filtered using 0.25 μm Millipore syringe filters into the autosampler vials. High-performance liquid chromatography (HPLC) was performed using Shim-pack XR-ODS column (3.0 mm *i*.*d*. and 100 mm length). Samples were resolved using a mobile phase consisting of a mixture of two solvents–solvent A (1.5% phosphoric acid) and solvent B (100% acetonitrile), taking 1.0 μl of sample as the injection volume. A 16 min linear gradient was applied at a flow rate of 0.7 ml/min and sinapine was detected via the UV/vis detector at 330 nm.

## Results

### Isolation of *SGT* and *SCT* genes from *B*. *juncea*

SGT has been reported to be an intronless gene in brassicas [21]. Hence, the full length *SGT* gene from *B*. *juncea* cv. Varuna was isolated from the genomic DNA through PCR amplification. A single band of ~1.5 kb was amplified and subsequent cloning and sequencing revealed a single sequence of 1494 bp. BLAST search of the sequence against the *B*. *juncea* genome sequence revealed the presence of four paralogs in the *B*. *juncea* genome [22] and showed sequence identity ranging from 99% to 89%. The identification of single paralog for the *SGT* gene in the present study could be due to the use of conserved primer for the amplification of the gene. This isolated *SGT* paralog (*BjSGT*) showed 99% of sequence identity with one of two functional paralogs of *B*. *napus SGT* gene [11] (**S2 Appendix, S2 Table**) and hence was used for the construction of different suppression constructs.

The *SCT* gene in related *Brassica* species and *Arabidopsis* contains 13 introns and 14 exons [23]. Hence, the coding sequence of the gene from *B*. *juncea* cv. Varuna was amplified from cDNA. PCR amplification from the cDNA and subsequent cloning and sequencing identified two paralogs in *B*. *juncea* one with 1401bp (named as *BjSCTCDS1*) and the other with 1410 bp (*BjSCTCDS2*). These two paralogs showed high sequence identity with the *SCT* cDNA paralogs of *B*. *napus*, *B*. *rapa* and *B*. *oleracea* (**S3 Appendix, S2 Table**). For the development of suppression constructs, cDNA sequence of *BjSCTCDS2* paralog was used.

### *In silico* analyses of three-dimensional structure and substrate interactions of SGT and SCT enzymes

**Fig 1:**
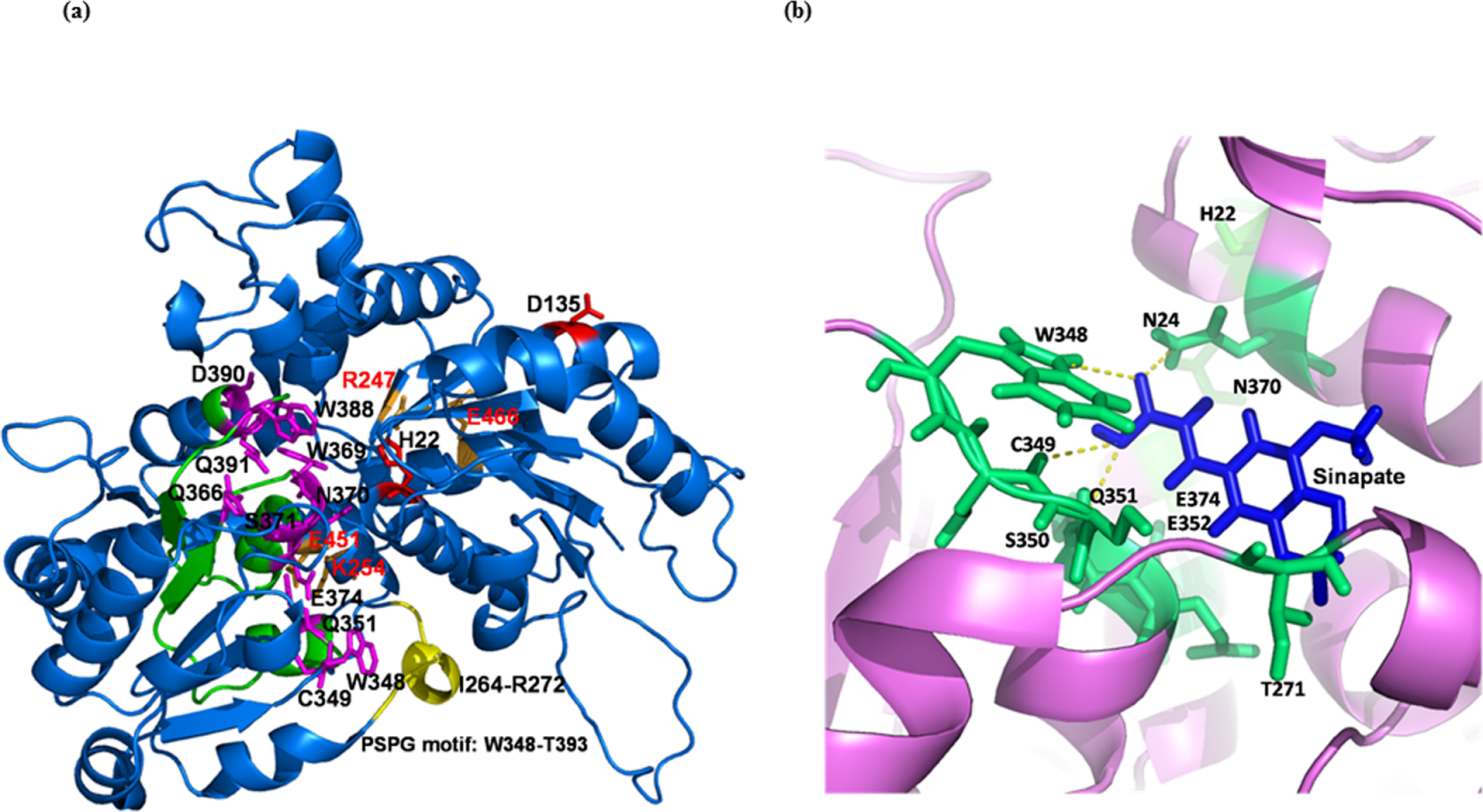
Three dimensional structure and binding mode of sinapate with SGT enzyme. (a) Three dimensional structure of SGT (blue cartoon format), where PSPG motif (W348-T393) is shown in green and flexible loop (I264-R272) in yellow. The functionally known residues of PSPG motif are highlighted in magenta sticks and two acceptor substrate binding residues are shown in red sticks. Residues forming salt bridges (R247-E466 and K254-E451) are shown in wheat colour sticks and labelled by red. (b) The binding mode of sinapate in deep cavity of SGT enzyme. The enzyme molecule is represented by pink colour cartoon and the sinapate molecule shown as blue stick. The hydrogen bonded residues are connected through the yellow dotted lines and represented in green sticks. Other than the hydrogen bonded residues, figure also shows other additional residues which also directly interact with sinapate by electrostatic interactions and Van der Waals forces.

The paralogs of *SGT* (*BjSGT*) and *SCT* (*BjSCTCDS2*) genes used for transgenic development were further assessed by *in-silico* analysis of their respective enzyme through three-dimensional structure and substrate interaction. The structural analysis of SGT enzyme revealed that both the N and C-terminal domains have six-stranded β- sheets flanked by eight and nine α-helices, respectively. The two domains are packed very compactly and form the binding site. Salt bridges between the residue R247-E466 and K254-E451 impart stability to the binding site. Highly conserved signature of putative secondary plant glycosyltransferase (PSPG) motif was observed at the C-terminal domain (**Fig 1A**).

SGT and sinapate interaction suggest that sinapate binds to the deep cavity of the active site of SGT via hydrogen bonding with W348, C349 and Q351 residues of PSPG motif (**Fig 1B**). The other residues of PSPG motif, S350, E352 and E374 interacted with sinapate by electrostatic interactions and Van der Waals forces. Furthermore, the N-terminal domain N24 and H22 of acceptor substrate binding site also interact with sinapate via hydrogen bond and electrostatic interactions. Thus, the interaction analyses suggested that SGT has high binding affinity with sinapate.

**Fig 2:**
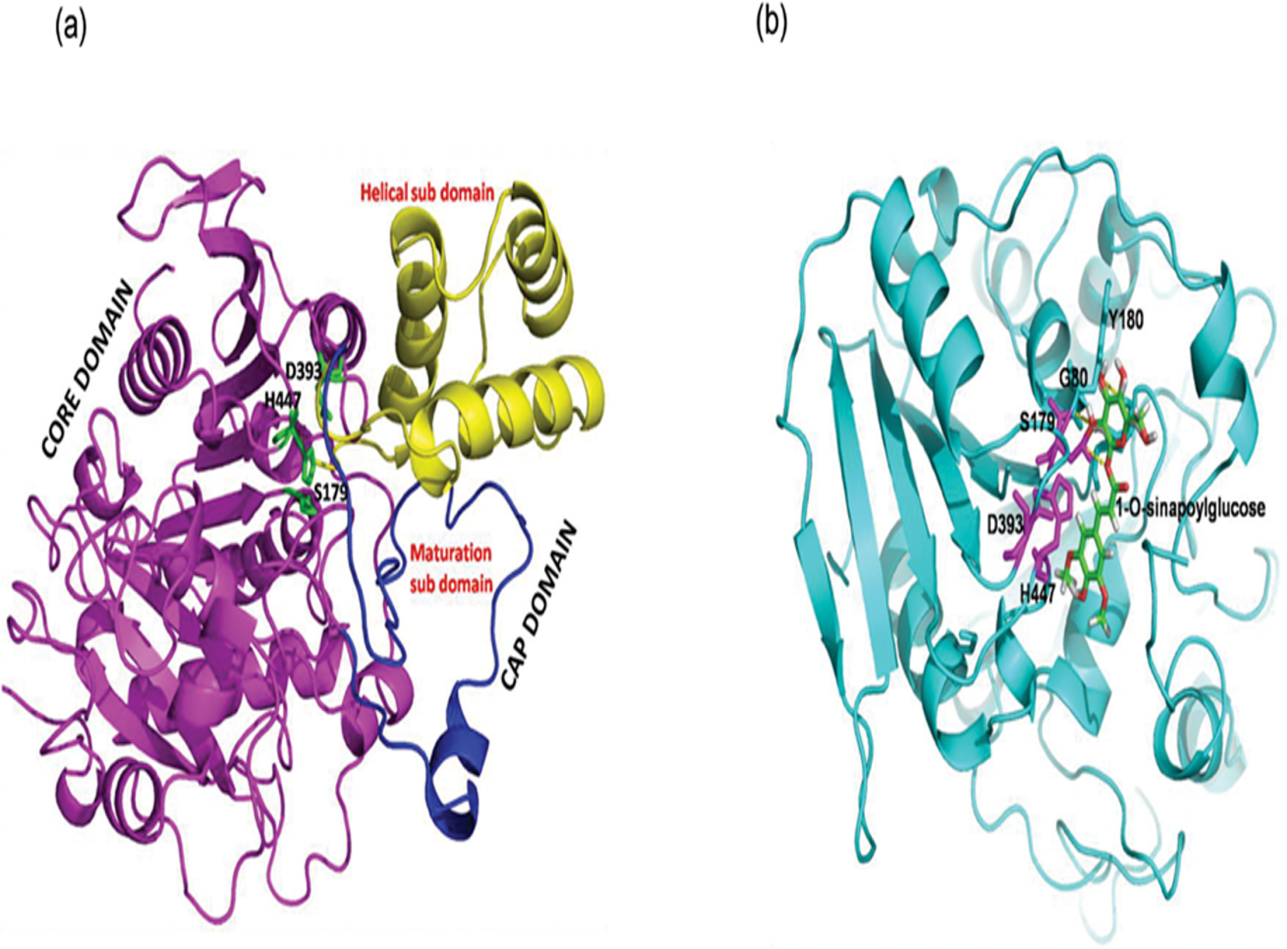
Three dimensional structure and binding of 1-*O*-sinapoylglucose with SCT enzyme. (a) Three dimensional structure of SCT enzyme. The core domain is shown in magenta, helical sub-domain in yellow and the maturation sub-domain is highlighted in dark blue. The catalytic triad residues are shown in green sticks. (b) The binding mode of 1-O-sinapoylglucose in deep cavity of SCT enzyme. The substrate 1-*O*-sinapoylglucose is represented by green stick whereas the cavity of the SCT is shown in cyan colour cartoon. The catalytic triad residues are shown as red sticks. The substrate molecule is hydrogen bonded (yellow dotted lines) with S179, Y180 and G80.

The SCT enzyme structural study showed that it contains a core and a cap domain. Structurally, the core domain (central β-sheet) is flanked by alpha helices on both the sides and harbour catalytic triad (Serine, Aspartate and Histidine) (**Fig 2A**). The cap domain is divided into a helical and a maturation sub domain. Structural analysis suggested that the maturation sub domain play a crucial role in the catalytic mechanism.

The docking analysis revealed that 1-*O*-sinapoylglucose interaction with the core domain (active site) of SCT enzyme is obstructed by the maturation subdomain (lys 282 –ser 320). Hence, there is a requirement of some conformational changes, or the excision of full or part of the maturation subdomain for binding of substrate to the active site. After removal of the maturation subdomain from SCT enzyme, 1-*O*-sinapoylglucose interacts with the active site of SCT which is situated deep inside (**Fig 2B**), and forms hydrogen bonds with catalytic residue S179 and two other G80 and Y180 residues. The catalytic residues D393 and H447 also interact with substrate by electrostatic interactions and Van der Waals forces. The docking study, therefore, suggested efficient binding affinity between SCT enzyme and 1-*O*-sinapoylglucose.

### Expression analysis of *SGT* and *SCT* genes in *B*. *juncea*

**Fig 3:**
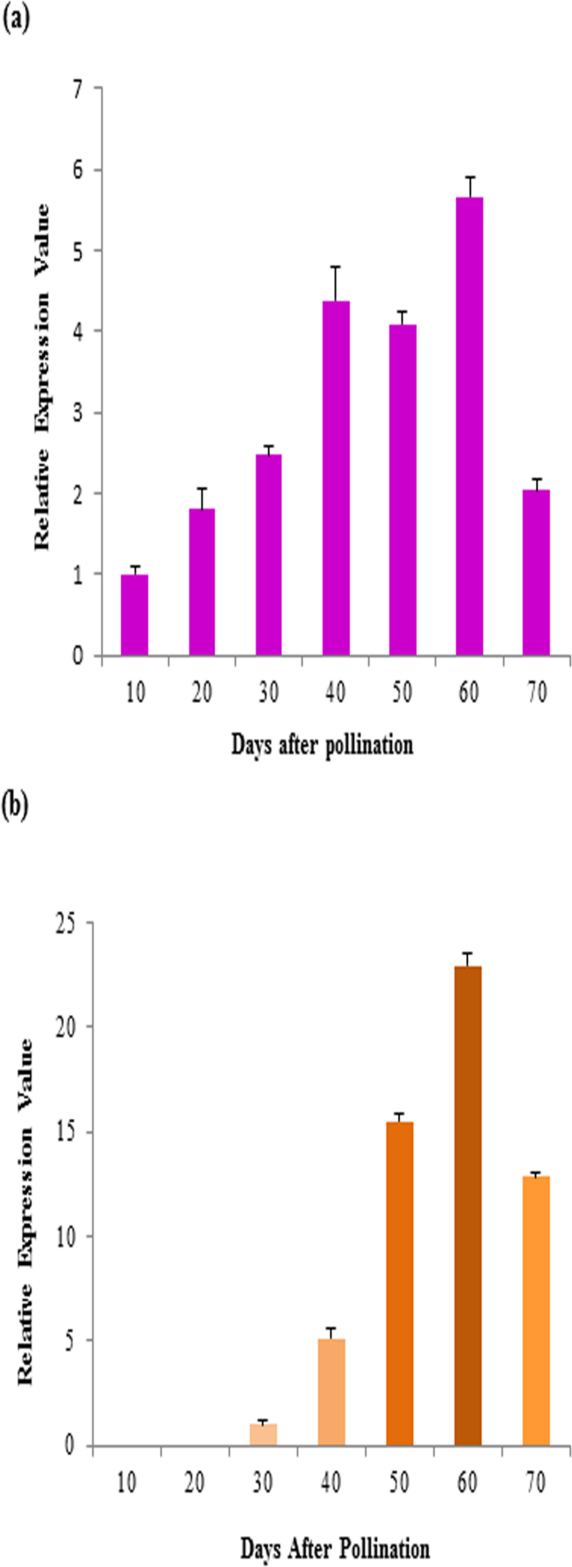
Expression analyses of (a) *SGT* and (b) *SCT* gene at different developmental stages of the seeds (10 to 70 DAP) of *B*. *juncea* cv. Varuna. The data are average of three biological replicates (±SD).

The expression of both *SGT* and *SCT* genes was measured at different stages of seed development in the wild type *B*. *juncea* cv. Varuna. This exercise was undertaken to find out the onset of expression of the two genes in order to decide the type of promoters to be used for effective suppression of these genes. Controlled self-pollination was performed and developing pods were harvested at 10, 20, 30, 40, 50, 60, and 70 days after pollination (DAP). Quantitative RT-PCR (qTR-PCR) detected accumulation of *SGT* transcripts from 10 DAP which continued till 70 DAP with peak levels at 40 to 60 DAP (**Fig 3A**). In case of *SCT* gene, the accumulation of transcripts was observed from 30 DAP with peak levels at 50 to 60 DAP (**Fig 3B**).

### Promoters used in the present study

For the down-regulation of the *SGT* gene, a minimal region of 350 bp of *napin* promoter [19] was amplified, sequenced and analyzed in Plant *cis*-regulatory DNA element database (*PLACE*). The primers used are given in **S1 Table**, Analysis revealed that promoter sequence contains different common regulatory elements as TATA and CAAT boxes, and also has seed-specific motifs (**S2A Fig**).

For the down-regulation of the *SCT* gene, endogenous promoter of highly expressing *BjSCTCDS1* paralog was used. The promoter was isolated through 5’ genome-walking. The 771 bp fragment from *Dra* I library was sequenced and analyzed in Plant *cis*-regulatory DNA element database (*PLACE*) and it was observed that the fragment contained different seed-specific and other common motifs (**S2B Fig**). A 750 bp upstream fragment (-1 to -750) was used as the promoter sequence in the suppression constructs for *SCT* gene.

### Development of transgenics in *B*. *juncea*

A total of 1549 putative independent transgenics (T_0_) from eight constructs were transferred to the containment net house during mustard growing season (**Table 1**). Coating the leaves of these putative transgenics with basta solution identified a total of 1232 basta resistant plants. The presence of *bar* gene was confirmed by PCR amplification of 550 bp fragment from randomly selected 20 basta resistant transgenics. All the 1232 basta resistant T_0_ plants were selfed and seeds (T_1_ seeds) were harvested separately from 992 healthy independent transformants (**Table 1**).

**Table 1:** Details of putative T_0_ transgenic plants transferred to containment net house for advancement to T_1_ generation.

Sl no.	Construct Name	Number of putative transgenic plants (T_0_) transferred	Number of basta resistant plants obtained	Number of T_0_ plants from which T_1_ seeds harvested	Number of T_1_ seed samples analyzed for sinapine content
**1**	BjSGTAS	217	153	124	56
**2**	BjSGTRNAi	55	48	45	43
**3**	BjSGTamiR38	240	179	148	60
**4**	BjSGTamiR40	217	189	167	135
**5**	BjSCTAS	127	107	89	79
**6**	BjSCTRNAi	130	82	63	33
**7**	BjSCTamiR36	281	231	193	56
**8**	BjSCTamiR37	282	243	163	62

### Genetic and molecular characterization of T_1_ transgenics

T_1_ seeds of 524 independent transformants (**Table 1**) that produced sufficient seeds (>500 seeds per line) were selected for genetic analysis. The seed sinapine content of these 524 independent T_1_ lines obtained using different constructs was analyzed by HPLC. The analysis showed that the seed sinapine content varied from 4.47 to 13.07 mg/g DSW (**S3 Table**). A total of 82 T_1_ lines, generated from six of the eight constructs (**S4 Table**) showing ≥ 30% reduction in seed sinapine content compared to that in the wild type genotype (Varuna), were selected for further analysis. None of the transgenic T_1_ lines obtained using BjSGTamiR38 and BjSCTamiR36 constructs showed ≥30% reduction in seed sinapine content (**S3 Table**).

A comparative analysis of the efficiency of the different constructs (antisense, RNAi and artificial micro RNA) as well as the relative importance of the targeted genes (*SGT* and *SCT*) in achieving better suppression of the seed sinapine based on the data obtained from T_1_ seeds was undertaken. The analysis indicated that suppression by RNAi was better than the other two constructs. 43.4% of the transgenic lines, obtained using RNAi construct, showed ≥30% reduction in the sinapine content vis-a-vis the wild type, as against 10.4% and 11.2% reduction in the lines obtained using antisense RNA and artificial micro RNA constructs, respectively (**Table 2**). The above observations were confirmed statistically. Paired t-test between the means revealed that all the three methods of suppression are significantly different from each other and confirmed that RNAi worked better than other two methods (**Table 2**).

**Table 2:** Summary of analysis of seed sinapine content of 524 T_1_ lines obtained using different suppression constructs against the target genes, highlighting the lines showing ≥ 30% reduction (≤ 8.19 mg/g DSW) compared to the wild type line, Varuna (11.70 ± 0.55 mg/g DSW).

Gene	Antisense RNA			RNAi			Artificial microRNA			Total	
	No. of lines analysed	Lines Showing ≥30% reduction		No. of lines analysed	Lines Showing ≥30% reduction		No. of lines analysed	Lines Showing ≥30% reduction			
		Number	Percentage		Number	Percentage		Number	Percentage	Number (mean sinapine content; mg/g DSW)	Percentage
**SGT**	56	6	10.7	43	28	65.1	195	34	17.4	**68/294** (9.42*)	**23.1**
**SCT**	79	8	10.1	33	5	15.2	118	1	0.84	**14/230** (9.80*)	**6.1**
**Total**	135	14	10.4	76	33	43.4	313	35	11.2	**82/524**	**15.6**
**(mean sinapine content; mg/g DSW)**	(9.38*)	**(7.72)**		(8.37*)	**(7.33)**		(9.98*)	**(7.21)**		**(9.60)**	

Paired t test between the means p value

Antisense RNA vs RNAi 1.09x10^-9^

Antisense RNA vs artificial micro-RNA 1.23x10^-5^

RNAi vs artificial micro-RNA 3.62x10^-18^

*SGT* vs *SCT* 0.0022.

*Significantly different.

A comparison between the two targeted genes for the suppression revealed that targeting *SGT* was more effective than targeting *SCT* gene in reducing sinapine levels. Whereas 23.1% of the transgenic lines obtained through the suppression of *SGT* showed ≥ 30% reduction in sinapine content than that in the wild type, only 6.1% of the lines yielded comparable result by targeting *SCT* gene. Statistical analysis by paired t-test between the means also confirmed the above observation (**Table 2)**.

T_1_ seeds of these above mentioned selected 82 lines were subjected to segregation analysis for the identification of the lines with single gene inheritance. Forty five to 85 T_1_ seeds from each independent line were sown in plant growth chambers. A transgenic *B*. *juncea* line, homozygous for basta resistance and available in the lab, was used as the positive control. One week after germination, the seedlings were sprayed with basta (200mg/l) and the number of resistant and sensitive plants was recorded after one week of spraying. A total of 21 lines obtained using the six constructs showed single-gene inheritance (showed 3:1 segregation as revealed by χ^2^ test) (**Table 3**). Remaining lines showed multi-gene inheritance. The balance seeds of these 21 lines showing single-gene inheritance were sown in rows in containment net house. All the 21 lines showed segregation for basta resistance when sprayed with basta (200mg/l) after one month of sowing. Ten basta resistant plants from each of the 21 lines were selfed and T_2_ seeds from 210 progeny plants of 21 T_1_ lines were harvested separately.

**Table 3:** Segregation data of 21 T_1_ lines showing single gene inheritance for basta resistance.

S. No.	Name of the constructs	Name of T_1_ transgenic line	Segregation data			
			Germinated seeds	R*	S*	χ^2^-value
1	BjSGTAS	BjSGTAS.4	70	54	16	0.17
		BjSGTAS.81	60	42	18	0.80
2	BjSGTRNAi	BjSGTRNAi.17	69	51	18	0.04
		BjSGTRNAi.22	50	34	16	1.30
		BjSGTRNAi.26	50	35	15	0.7
		BjSGTRNAi.38	50	36	14	0.2
		BjSGTRNAi.53	45	35	10	0.2
3	BjSGTamiR40	BjSGTamiR40.3	48	35	13	0.1
		BjSGTamiR40.35	50	39	11	0.2
		BjSGTamiR40.42	50	35	15	0.7
		BjSGTamiR40.67	46	37	9	0.7
		BjSGTamiR40.77	50	41	9	1.3
		BjSGTamiR40.89	25	16	9	1.6
4	BjSCTAS	BjSCTAS.8	48	38	10	0.4
		BjSCTAS.36	51	33	18	2.9
		BjSCTAS.45	50	38	12	0.0
		BjSCTAS.81	40	30	10	0.0
5	BjSCTRNAi	BjSCTRNAi.1	65	55	10	3.21
		BjSCTRNAi.36	55	36	19	2.67
		BjSCTRNAi.52	72	61	11	3.62
6	BjSCTamiR37	BjSCTamiR37.56	58	44	14	0.02
	Line with multi-gene inheritance	BjSGTAS.59	80	80	0	26.67

*R: resistant to basta; S: sensitive to basta.

Of the 21 lines showing single-gene inheritance, DNA of 2 lines each from six different constructs were subjected to PCR amplification for the presence of the transgenes. All the lines amplified the expected fragment and confirmed the presence of the transgenes and their stable integration into the genome (**S3 Fig**).

Eleven basta resistant T_1_ lines showing single gene inheritance were selected randomly along with a T_1_ line showing multi-gene inheritance (BjSGTAS.59) and were subjected to southern hybridization to determine the number of transgene inserts in these events. Wild type Varuna was used as the control. DNA was digested with *Eco* RI (having one restriction site in the constructs) and was hybridized to the *bar* gene (**S1 Fig**). The analysis revealed that seven out of 11 lines showing single-gene inheritance had single-copy integration while the remaining four had two-copy integration. The multi-gene inheritance line (BjSGTAS.59) showed three-copy integration (**S4 Fig**).

**Fig 4:**
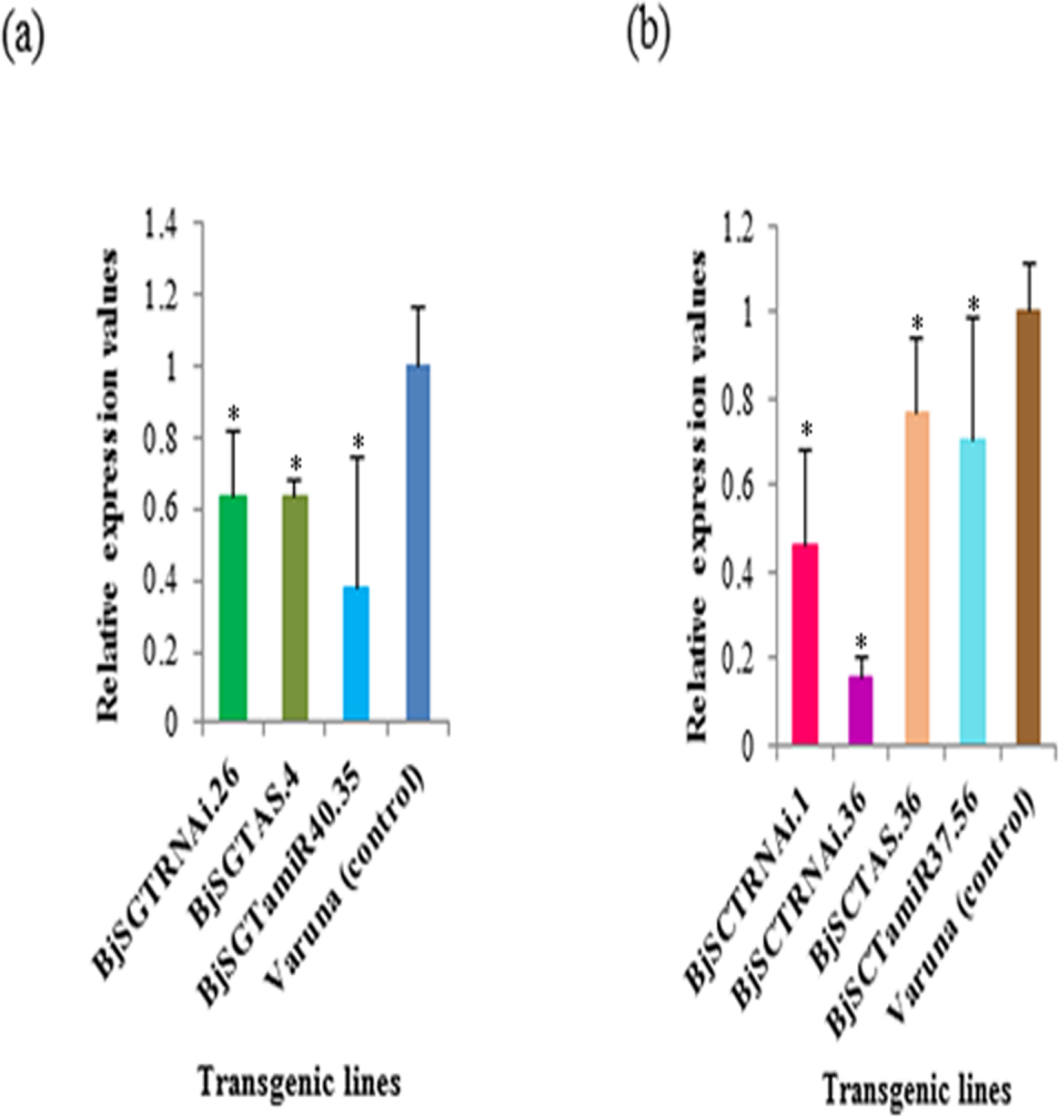
qRT-PCR analysis of (a) *SGT* and (b) *SCT* gene for transcript accumulation in T_1_ transgenic lines developed from different silencing constructs along with wild type, Varuna. Data are average of three biological replicates (±SE). Significant difference (*) was calculated at p < 0.05 (Fisher’s LSD test).

Expression analysis was undertaken using seven randomly selected basta resistant T_1_ lines (three derived using *SGT* and four using *SCT* constructs) showing single-gene inheritance along with wild type control. RNA was isolated from the developing seeds (40 DAP) generated by controlled self- pollination and was subjected to qRT-PCR analysis. The expression level of all the representative transgenic lines showed lowering of transcript level compared to that in the wild type control. The level of reduction for the *SGT* transcript varied from 1.3 to 2.6 fold. The lowest expression was observed in the line obtained using BjSGTamiR40.35 (**Fig 4A**). The reduction in *SCT* gene transcript ranged from 1.2 to 6.3 fold, the lowest being reported in the line developed using BjSCTRNAi.36 (**Fig 4B**). However, the expression study showed weak correlation between expression at RNA level and the reduction in the sinapine content.

### Genetic analysis of T_2_ transgenics and establishment of homozygous lines

T_2_ seedlings of 210 progeny plants were studied for segregation following the procedure used for the analysis of T_1_ seedlings. Twenty five of these 210 lines showed no segregation for basta resistance (all plants were basta resistant) and, hence, were identified as homozygous lines (**Table 4**). Analysis of variance (ANOVA) undertaken for the sinapine content of these 25 homozygous lines along with wild type (Varuna) indicated that all the transgenic lines had significantly lower sinapine content than the wild type line Varuna (**Table 4**). Seed sinapine content of these homozygous lines varied from 9.73 mg/g DSW to 3.79 mg/g DSW. Percent reduction of sinapine ranged from 15.8% to 67.2%. Majority of the homozygous lines (14 out of 25) showed ≥ 30% reduction in sinapine content than the wild type line (**Table 4**), the best T_2_ homozygous plant (BjSGTamiR40.35.1) registering 67.2% reduction having seed sinapine content at 3.79 mg/g DSW.

**Table 4:** Seed sinapine content and the percent reduction in 25 homozygous T_2_ lines compared to the wild type line Varuna.

S. No.	Construct Name	T_1_ lines with single gene inheritance		Homozygous T_2_ lines		
		Number	Name	Name	Sinapine content (mg/g DSW)*	Percent reduction of Sinapine from Varuna
1	BjSGTAS	2	BjSGTAS.4	BjSGTAS.4.1	9.34 ± 0.26	19.2
			BjSGTAS.81	BjSGTAS.81.5	6.74 ± 0.05	41.7
2	BjSGTRNAi	5	BjSGTRNAi.17	BjSGTRNAi.17.4	8.20±0.77	29.1
			BjSGTRNAi.22	BjSGTRNAi.22.2	8.35 ± 0.83	27.8
				BjSGTRNAi.22.3	8.02 ± 0.72	30.6
			BjSGTRNAi.26	BjSGTRNAi.26.5	6.70 ± 0.47	42.0
			BjSGTRNAi.38	BjSGTRNAi.38.4	7.92 ± 0.92	31.5
				BjSGTRNAi.38.6	7.91 ± 1.19	31.6
			BjSGTRNAi.53	BjSGTRNAi.53.1	8.37 ± 0.48	27.6
3	BjSGTamiR40	6	BjSGTamiR40.3	BjSGTamiR40.3.6	7.31 ± 0.42	36.8
			BjSGTamiR40.35	BjSGTamiR40.35.1	3.79 ± 0.25	67.2
			BjSGTamiR40.42	BjSGTamiR40.42.1	6.02 ± 0.64	47.9
			BjSGTamiR40.67	BjSGTamiR40.67.2	7.98 ± 0.46	31.0
			BjSGTamiR40.77	BjSGTamiR40.77.2	8.00 ± 1.03	30.8
			BjSGTamiR40.89	BjSGTamiR40.89.4	8.55 ± 0.1	26.0
4	BjSCTAS	4	BjSCTAS.8	BjSCTAS.8.8	9.73±0.87	15.8
			BjSCTAS.36	BjSCTAS.36.4	8.40 ± 0.65	27.3
			BjSCTAS.45	BjSCTAS.45.6	6.60 ± 0.36	42.9
			BjSCTAS.81	BjSCTAS.81.8	8.13 ± 0.86	29.7
				BjSCTAS.81.9	8.66 ± 0.22	25.1
5	BjSCTRNAi	3	BjSCTRNAi.1	BjSCTRNAi.1.1	7.76 ± 0.99	32.9
				BjSCTRNAi.1.8	7.84 ± 0.22	32.2
			BjSCTRNAi.36	BjSCTRNAi.36.4	9.43 ± 0.11	18.4
			BjSCTRNAi.52	BjSCTRNAi.52.3	4.95 ± 1.10	57.2
6	BjSCTamiR37	1	BjSCTamiR37.56	BjSCTamiR37.56.1	8.25 ± 0.23	28.6
	Varuna (Control)				11.56 ± 0.22	0.0
	Total	21		25		

* CD at 1% level = 0.1952; CD at 5% level = 0.1459.

## Discussion

The present study was undertaken with the aim of reducing sinapine content from the seeds of *B*. *juncea* cv. Varuna using transgenic approach. In *B*. *napus*, Bhinu et al. (2009) showed that silencing of the genes involved in the terminal steps of the phenylpropanoid pathway is more efficient in down regulating sinapine content than down regulating the genes in the initial steps of the phenylpropanoid pathway [1]. Hence, two genes in the final steps of the phenylpropanoid pathway, *SGT* encoding the enzymes glucosyltransferase (UDP-glucose:sinapate glucosyltransferase) and *SCT* encoding the sinapoylglucose: choline sinapoyltransferase, were targeted for developing suppression constructs to reduce the sinapine content in *B*. *juncea*.

Prior to the development of the constructs, two target genes (*SGT* and *SCT*) were isolated from wild type *B*. *juncea*. The functionality of the gene paralogs used for transgenic development was predicted by *in-silico* analysis of their respective enzymes through three-dimensional structure and substrate interactions. The patterns of expression of *SGT* and *SCT* genes in the developing seeds of wild type *B*. *juncea* were studied for the selection of a suitable promoter that would optimally drive the expression of the transgenes. The investigation revealed an early onset of expression for the *SGT* gene (10 DAP stage) and a late onset of expression (30 DAP stage) for the *SCT* gene in *B*. *juncea* and showed a concurrence with an earlier study on the expression analyses of these two genes in *B*. *napus* [10].

The *SGT* and *SCT* genes are members of glucosyltransferase and serine carboxypeptidase like acyltransferase multigene families [24–25]. The manipulation of these genes should be controlled precisely so as to have minimal adverse effects on the plants through ectopic expressions. Instances of impairment of normal activity of plants as a result of manipulation of genes of multigene families have been reported earlier [26–27]. Constitutive promoters are known to produce off-target effects, which may be deleterious to the plants [28]. Since both *SGT* and *SCT* are expressed during seed development stages, seed specific promoters have been used in the present study for down regulating these two genes in order to avoid the possibilities of ectopic expression.

The rationale for using *napin* seed specific promoter in driving *SGT* gene was that firstly, the *napin* promoter shows early onset of expression [29] that overlaps with the expression of *SGT* gene and secondly, we did not have relative functionality data of different paralogs for isolating an endogenous promoter from a better expressing paralog as we could not isolate more than one functional paralog from the *SGT* gene of *B*. *juncea*. Conversely for the *SCT* gene, we could use the endogenous promoter of a better expressing paralog as we had the relative expression data of all the two functional paralogs.

It was observed that reduction in sinapine content in the transgenic lines was more when *SGT* gene was silenced. One of the reasons for this could be the use of different promoters for silencing the two genes. The *napin* promoter used for the suppression of the *SGT* gene could be more active than the endogenous promoter used for silencing the *SCT* gene. Our results also reveal that amongst the three types of constructs used, suppression of both *SGT* and *SCT* genes using RNAi constructs showed an overall higher impact. In a previous study sinapine ester content was shown to be reduced by 54% in *B*. *napus* by using an RNAi suppression construct against the *SGT* gene [2].

Artificial microRNA (amiRNA) mediated gene silencing is another potential tool for down regulation of endogenous genes in plants [30]. The primary benefit for adopting amiRNA technology is its specificity [31–32]. Artificial microRNA in plants reduces gene expression primarily by eliciting cleavage of homologous RNA transcripts. For effective application of amiRNAs, homology and the accessibility of the target sequence are of great importance [33–36]. Artificial microRNA mediated gene silencing in plants, are shown to be mostly effective in diploid species such as *Arabidopsis* [37], rice [38], eggplant [39] and tomato [40]. Application of artificial microRNA in polyploid plant species is very limited. In a polyploid species where more than one expressing paralogs are expected, an efficient silencing by amiRNA technology depends on the identification of an appropriate shared target sequence to silence all the paralogs. To pave the way for the efficient application of amiRNA technology in polyploid *Brassica* species, Dhakate et al. (2014) designed an amiRNA (amiR-bnashp2) based on the two homologs of *SHP1* and *SHP2* (SHATTERPROOF genes) from *B*. *napus* and showed its efficiency in redundantly silencing both *SHP1* and *SHP2* by detection of the cleavage products of *SHP1* and *SHP2* transcripts in *B*. *napus*, *B*. *juncea* and *Arabidposis* [41].

In the present study on *B*. *juncea*, a polyploid crop, four different amiRNA constructs (BjSGTamiR38, BjSGTamiR40, BjSCTamiR36 and BjSCTamiR37) were used for the suppression of *SGT* and *SCT* genes. The results showed that out of the four silencing constructs, two, namely BjSGTamiR40 and BjSCTamiR37, were more effective in silencing. This finding probably corroborates the previous observations [35–36] that the structure of the target and mRNA surrounding the target site could affect the efficiency of miRNA silencing. Moreover, the presence of more than one expressing paralogs in a polyploid might have also compromised the suppression of *SGT* and *SCT* genes using artificial microRNA. There are several aspects that are still unknown regarding the processing of plant miRNA precursors and/or the mechanisms by which microRNAs recognize their targets. It has been shown that not only the structure of the target [35] but also the structure of mRNA surrounding the target site also affect the efficiency of miRNA silencing in diploid animal models [36]. It appears that more research efforts are needed to optimize the application of amiRNA technology in polyploid plant species. However, the transgenic line showing maximum of 67.2% reduction in seed sinapine content in our study was from an artificial microRNA construct (BjSGTamiR40.35.1).

Some of the transgenic lines that were marked as showing single gene inheritance on the basis of segregation analysis were later shown to have multiple insertion of the transgene(s) through Southern hybridization. This could be due to multiple insertion of the transgene *en bloc*, leading to its inheritance as a single recombination block in the genetic analysis. The study also revealed variable degree of silencing among the different homozygous transgenic lines in T_2_ generation. It has been observed in many agronomically important crops, that transgenic lines developed with different silencing constructs show variability in the extent of silencing efficiency and consequent effects on the phenotype of these plants [28,42–43].

## Conclusions

In the present study, a large number of independent transgenic lines were developed using three types of suppression constructs to silence the two genes involved in the final two steps of sinapine biosynthetic pathway. Significant level of reduction in sinapine content (up to 67%) was achieved in seeds of transgenic *B*. *juncea* lines. Systematic genetic and molecular analyses were undertaken to identify low sinapine lines. The selection marker (*bar* gene) was cloned within *lox-P* site and hence, could be subsequently removed [44]. The low sinapine lines could either be used directly in future for improving the quality of seed meal as these low sinapine lines have comparable oil content with the wild type or could be used in the future breeding programme(s) for further reducing the sinapine content and improving the quality of seed meal in oilseed mustard *B*. *juncea*. For example, the line having 3.79 mg/g DSW of sinapine through suppressing *SGT* gene (BjSGTamiR40.35.1) could be crossed with another low sinapine line containing 4.95 mg/g DSW developed through suppressing *SCT* gene (BjSCTRNAi52.3) for further reducing the seed sinapine content.

## Supporting information

S1 Fig Map of T-DNA of eight different transformation constructs. (PPTX)

S2 Fig Schematic diagram showing different seed-specific motifs and common elements of *napin* and *SCT* gene endogenous promoters. (PPTX)

S3 Fig PCR amplification of the transgenes from representative T_1_ transgenic lines from different silencing constructs. (PPTX)

S4 Fig Southern analysis of representative T1 transgenic lines from different silencing constructs. (PPTX)

S1 Table Primer sequences of *SGT* and *SCT* genes used in the present study. (DOCX)

S2 Table Sequence identity of *SGT* and *SCT* genes with different *brassica* species. (DOCX)

S3 Table Seed sinapine contents (mg/g DSW) of T_1_ transgenics developed by eight suppression constructs in *B*. *juncea*. (DOCX)

S4 Table T_1_ lines showing ≥30% reduction in seed sinapine content than the wild type genotype (Varuna). (DOCX)

S1 Appendix Development of different transformation constructs for *SGT* and *SCT* genes. (DOCX)

S2 Appendix Full length *SGT* gene sequences of different *Brassica* species. (PPTX)

S3 Appendix CDS sequences of *SCT* gene homologs from *B*. *juncea* and other *Brassica* species. (PPTX)
