# Supplementary material for: Development of transgenic Brassica juncea lines for reduced seed sinapine content by perturbing phenylpropanoid pathway genes
Source: PLoS One. 2017 Aug 7;12(8):e0182747. doi: 10.1371/journal.pone.0182747 (PMC5546701; doi:10.1371/journal.pone.0182747)
Supplement: S1 Fig — (PPTX) [file pone.0182747.s001.pptx]

## Slide 1
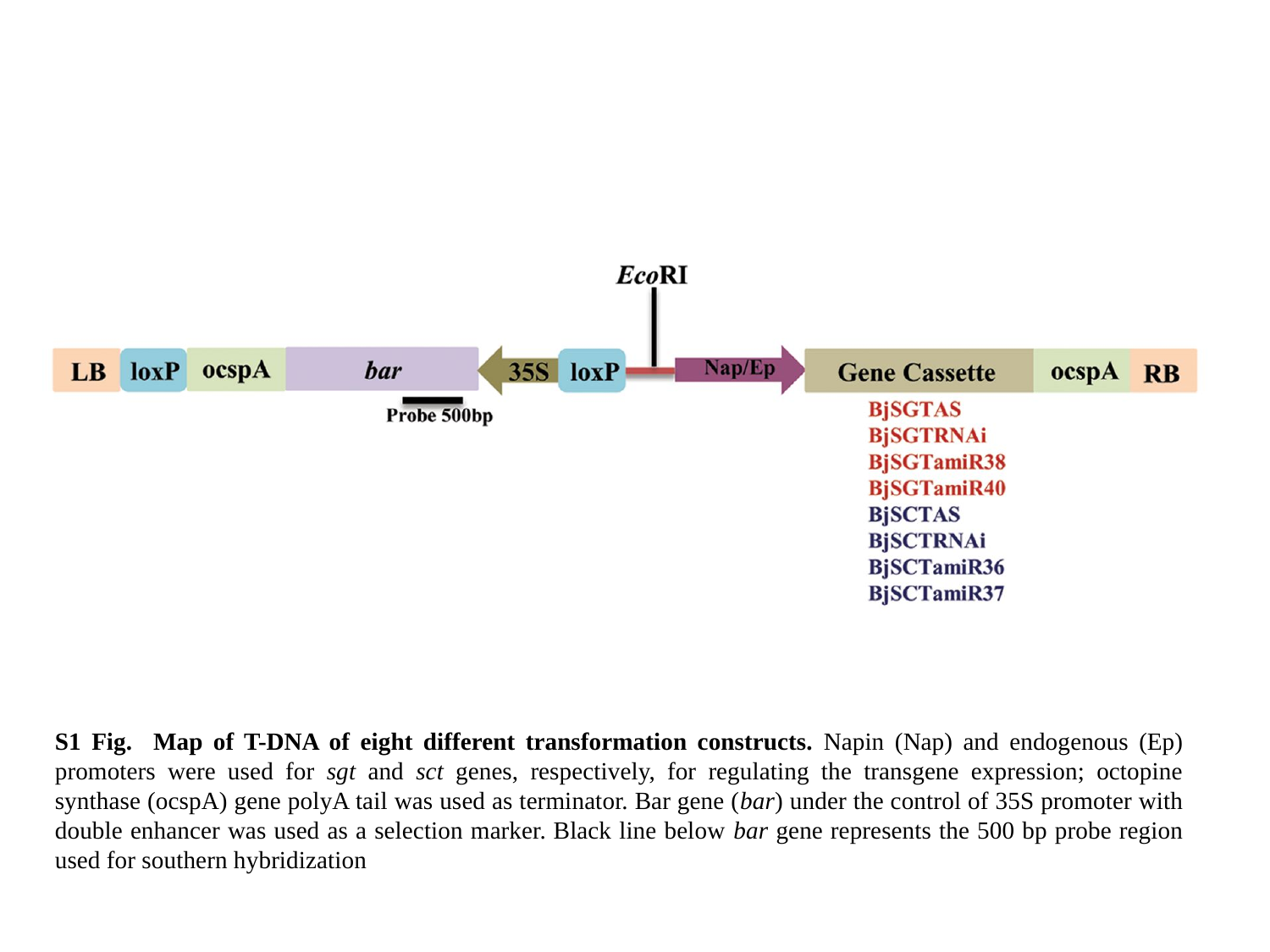

S1 Fig. Map of T-DNA of eight different transformation constructs. Napin (Nap) and endogenous (Ep) promoters were used for sgt and sct genes, respectively, for regulating the transgene expression; octopine synthase (ocspA) gene polyA tail was used as terminator. Bar gene (bar) under the control of 35S promoter with double enhancer was used as a selection marker. Black line below bar gene represents the 500 bp probe region used for southern hybridization
