# Supplementary material for: Development of transgenic Brassica juncea lines for reduced seed sinapine content by perturbing phenylpropanoid pathway genes
Source: PLoS One. 2017 Aug 7;12(8):e0182747. doi: 10.1371/journal.pone.0182747 (PMC5546701; doi:10.1371/journal.pone.0182747)
Supplement: S2 Fig — (PPTX) [file pone.0182747.s002.pptx]

## Slide 1
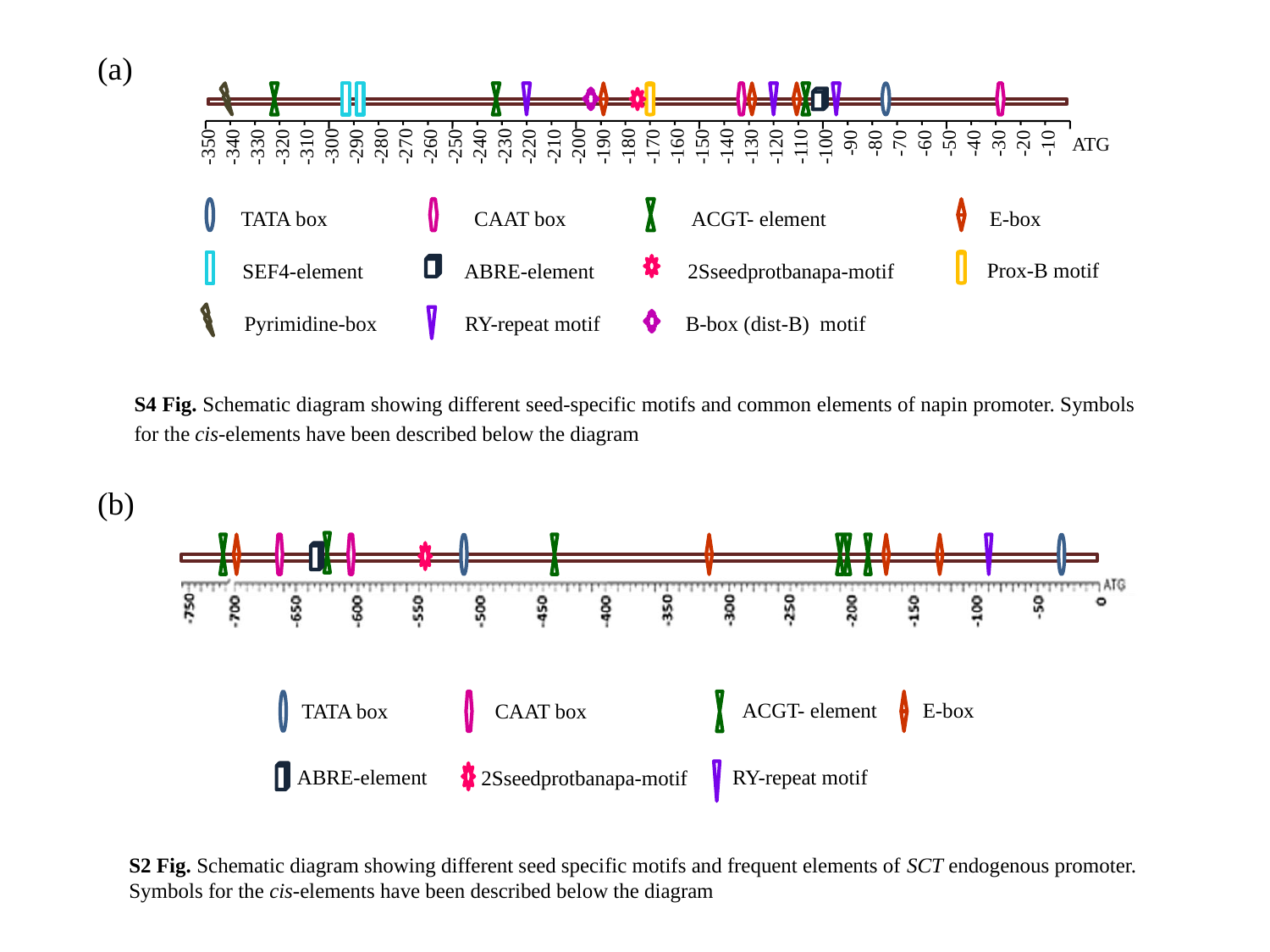

(a)
ACGT- element
E-box
TATA box
CAAT box
Prox-B motif
ABRE-element
SEF4-element
2Sseedprotbanapa-motif
Pyrimidine-box
RY-repeat motif
B-box (dist-B) motif
ATG
-90
-80
-70
-60
-50
-40
-30
-20
-10
-300
-290
-280
-270
-260
-250
-240
-230
-220
-210
-200
-190
-180
-170
-160
-150
-140
-130
-120
-110
-100
-350
-340
-330
-320
-310
S4 Fig. Schematic diagram showing different seed-specific motifs and common elements of napin promoter. Symbols for the cis-elements have been described below the diagram
(b)
ACGT- element
E-box
TATA box
CAAT box
ABRE-element
RY-repeat motif
2Sseedprotbanapa-motif
S2 Fig. Schematic diagram showing different seed specific motifs and frequent elements of SCT endogenous promoter. Symbols for the cis-elements have been described below the diagram
