# Supplementary material for: Development of transgenic Brassica juncea lines for reduced seed sinapine content by perturbing phenylpropanoid pathway genes
Source: PLoS One. 2017 Aug 7;12(8):e0182747. doi: 10.1371/journal.pone.0182747 (PMC5546701; doi:10.1371/journal.pone.0182747)
Supplement: S3 Fig — (PPTX) [file pone.0182747.s003.pptx]

## Slide 1
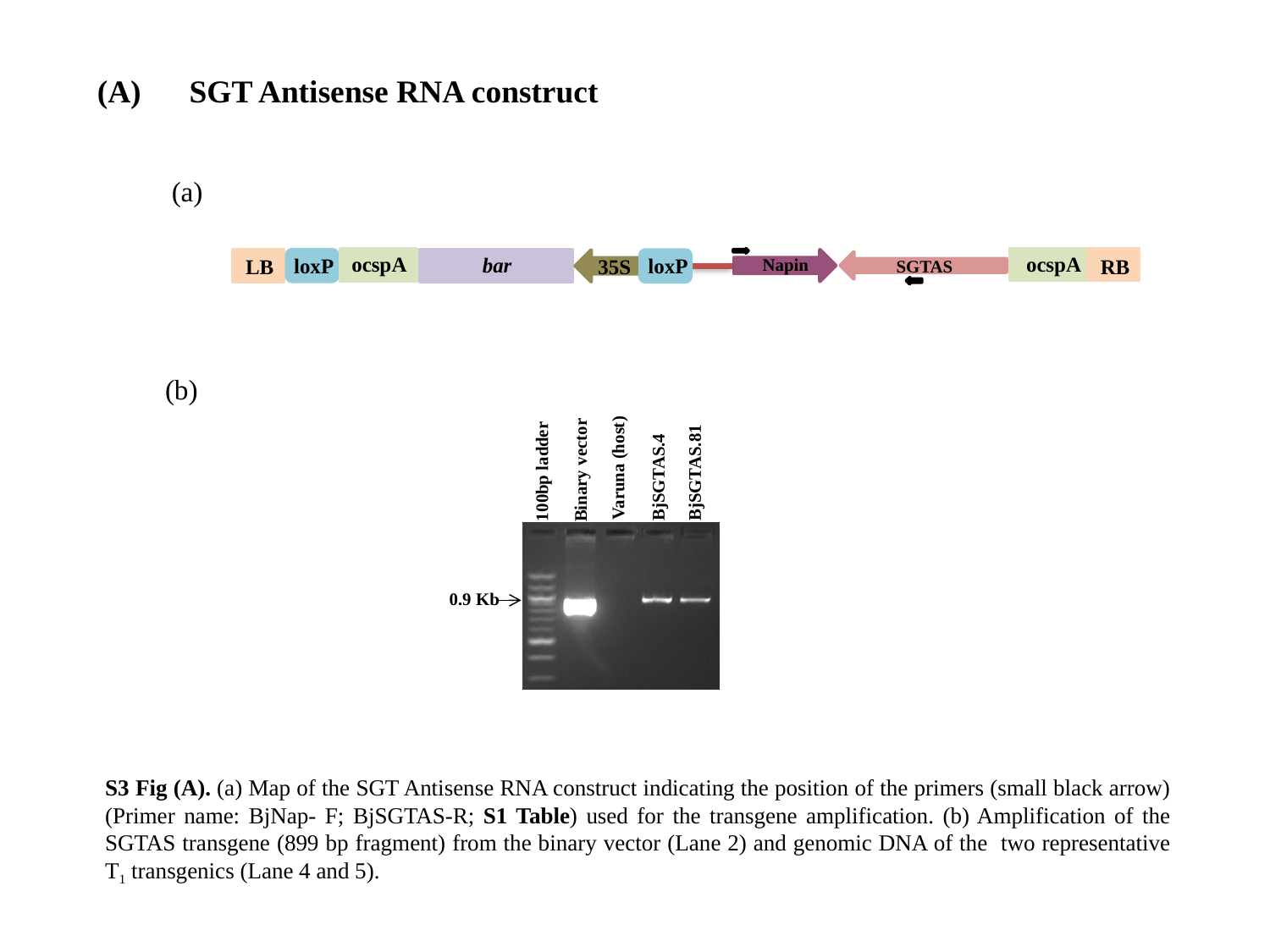

(A) SGT Antisense RNA construct
(a)
ocspA
bar
loxP
loxP
Napin
LB
35S
ocspA
RB
SGTAS
(b)
Varuna (host)
Binary vector
100bp ladder
BjSGTAS.81
BjSGTAS.4
0.9 Kb
S3 Fig (A). (a) Map of the SGT Antisense RNA construct indicating the position of the primers (small black arrow) (Primer name: BjNap- F; BjSGTAS-R; S1 Table) used for the transgene amplification. (b) Amplification of the SGTAS transgene (899 bp fragment) from the binary vector (Lane 2) and genomic DNA of the two representative T1 transgenics (Lane 4 and 5).

## Slide 2
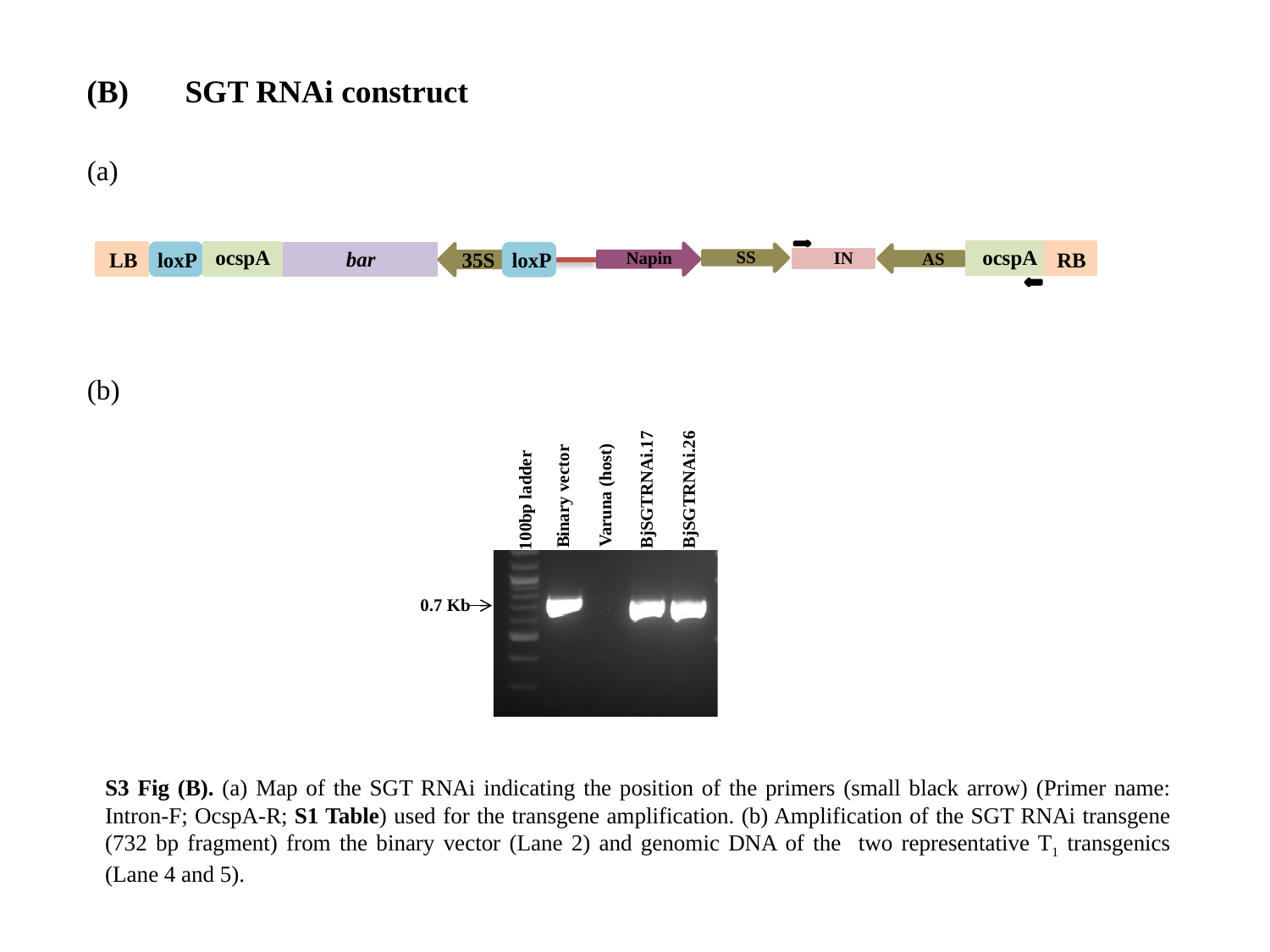

(B) SGT RNAi construct
(a)
ocspA
bar
loxP
loxP
Napin
LB
35S
ocspA
RB
SS
IN
AS
(b)
BjSGTRNAi.26
BjSGTRNAi.17
Varuna (host)
Binary vector
100bp ladder
0.7 Kb
S3 Fig (B). (a) Map of the SGT RNAi indicating the position of the primers (small black arrow) (Primer name: Intron-F; OcspA-R; S1 Table) used for the transgene amplification. (b) Amplification of the SGT RNAi transgene (732 bp fragment) from the binary vector (Lane 2) and genomic DNA of the two representative T1 transgenics (Lane 4 and 5).

## Slide 3
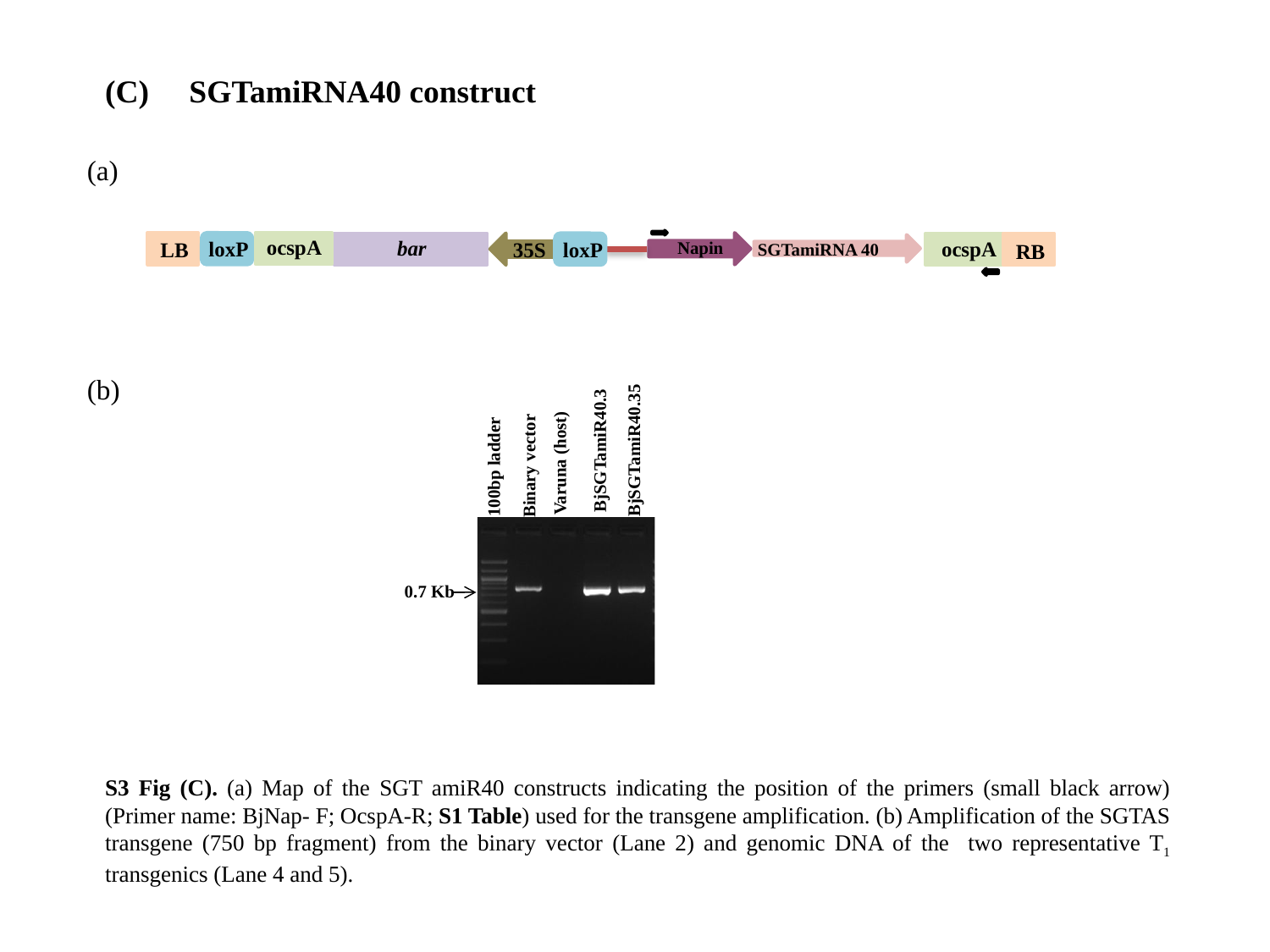

(C) SGTamiRNA40 construct
(a)
ocspA
bar
loxP
loxP
Napin
LB
35S
ocspA
RB
SGTamiRNA 40
(b)
BjSGTamiR40.35
BjSGTamiR40.3
Varuna (host)
Binary vector
100bp ladder
0.7 Kb
S3 Fig (C). (a) Map of the SGT amiR40 constructs indicating the position of the primers (small black arrow) (Primer name: BjNap- F; OcspA-R; S1 Table) used for the transgene amplification. (b) Amplification of the SGTAS transgene (750 bp fragment) from the binary vector (Lane 2) and genomic DNA of the two representative T1 transgenics (Lane 4 and 5).

## Slide 4
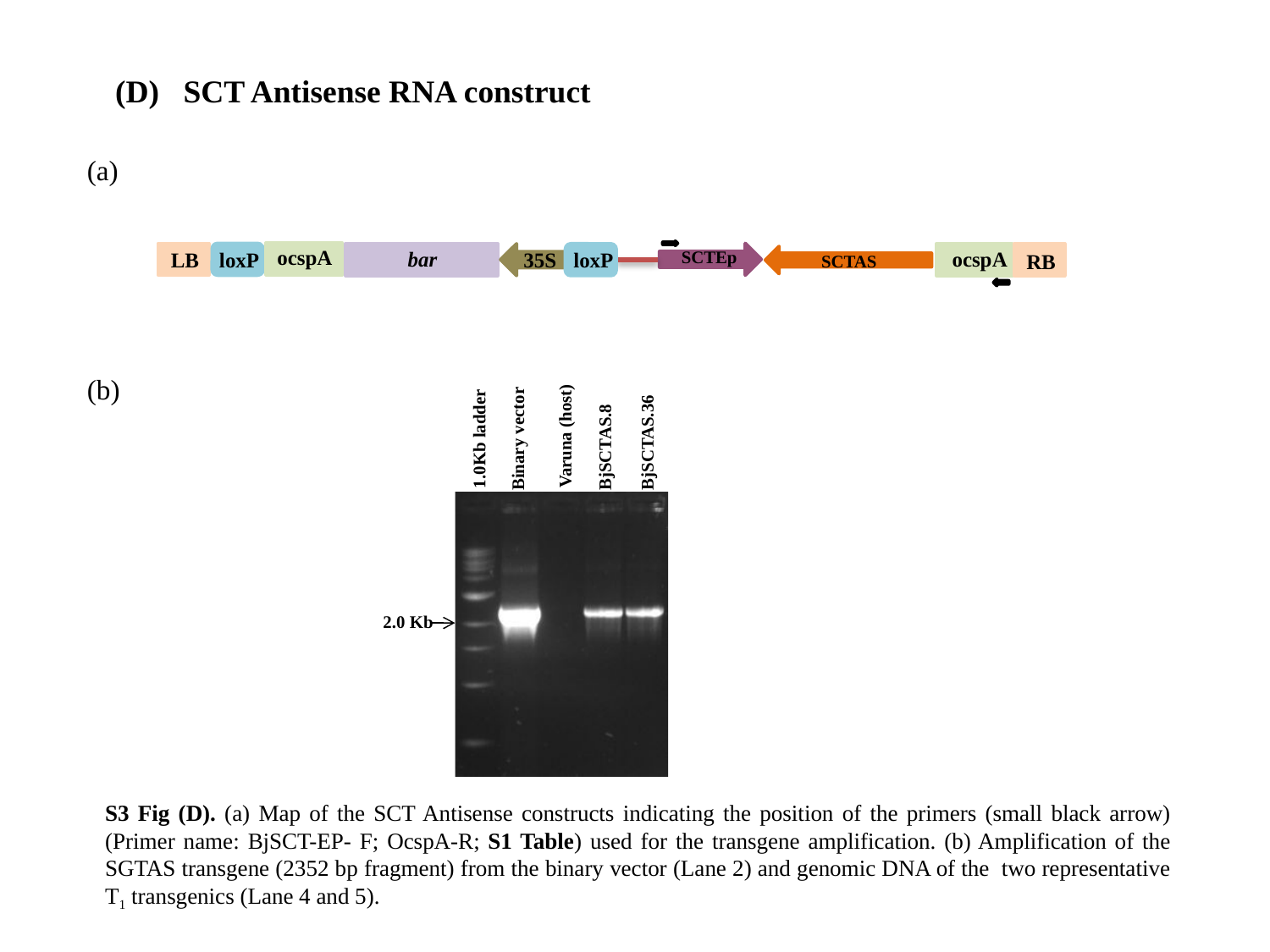

(D) SCT Antisense RNA construct
(a)
ocspA
bar
SCTEp
loxP
loxP
LB
35S
ocspA
RB
SCTAS
(b)
Varuna (host)
Binary vector
1.0Kb ladder
BjSCTAS.36
BjSCTAS.8
2.0 Kb
S3 Fig (D). (a) Map of the SCT Antisense constructs indicating the position of the primers (small black arrow) (Primer name: BjSCT-EP- F; OcspA-R; S1 Table) used for the transgene amplification. (b) Amplification of the SGTAS transgene (2352 bp fragment) from the binary vector (Lane 2) and genomic DNA of the two representative T1 transgenics (Lane 4 and 5).

## Slide 5
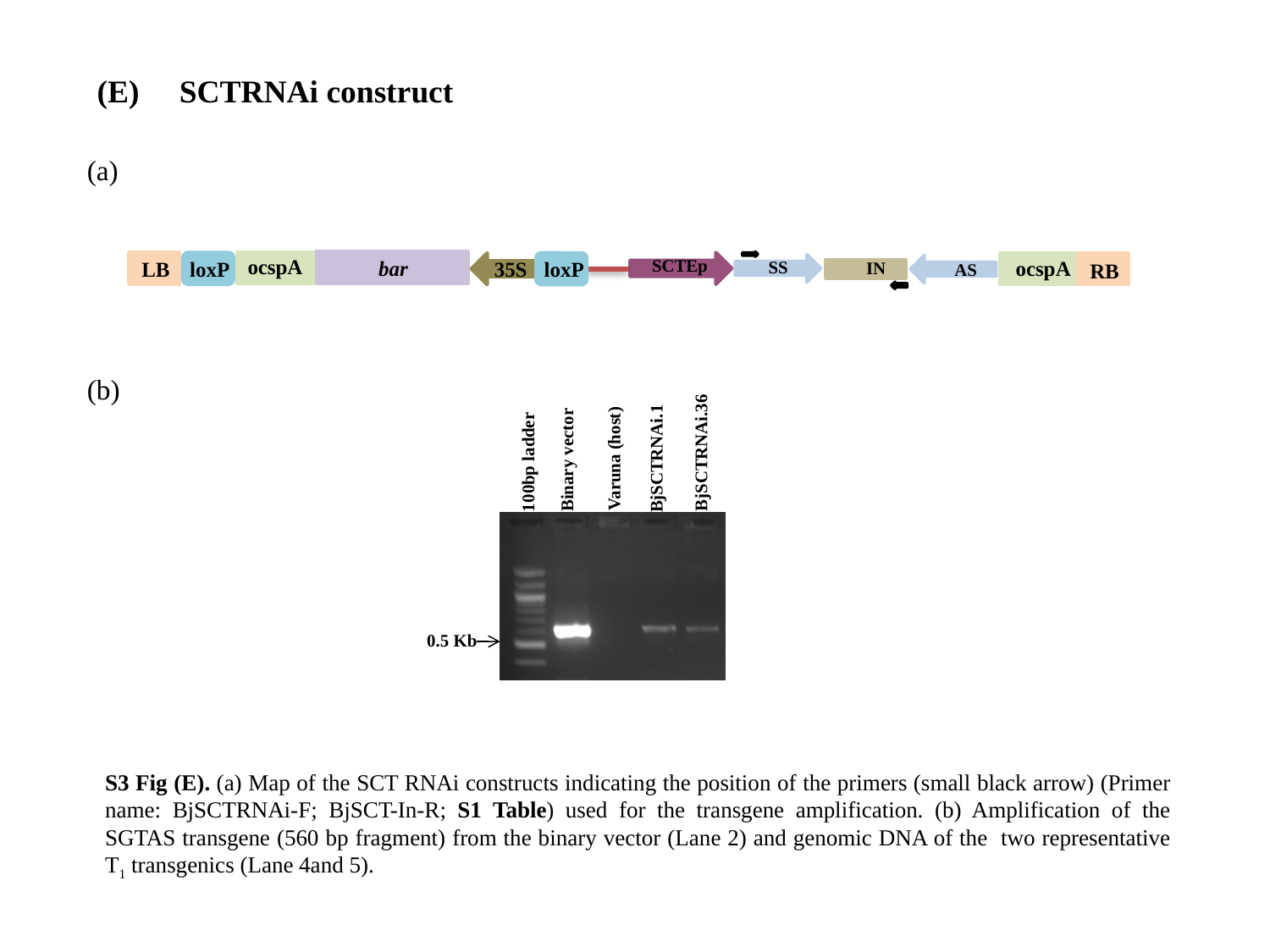

(E) SCTRNAi construct
(a)
ocspA
bar
SCTEp
loxP
loxP
LB
35S
ocspA
RB
SS
IN
AS
(b)
BjSCTRNAi.36
BjSCTRNAi.1
Varuna (host)
Binary vector
100bp ladder
0.5 Kb
S3 Fig (E). (a) Map of the SCT RNAi constructs indicating the position of the primers (small black arrow) (Primer name: BjSCTRNAi-F; BjSCT-In-R; S1 Table) used for the transgene amplification. (b) Amplification of the SGTAS transgene (560 bp fragment) from the binary vector (Lane 2) and genomic DNA of the two representative T1 transgenics (Lane 4and 5).

## Slide 6
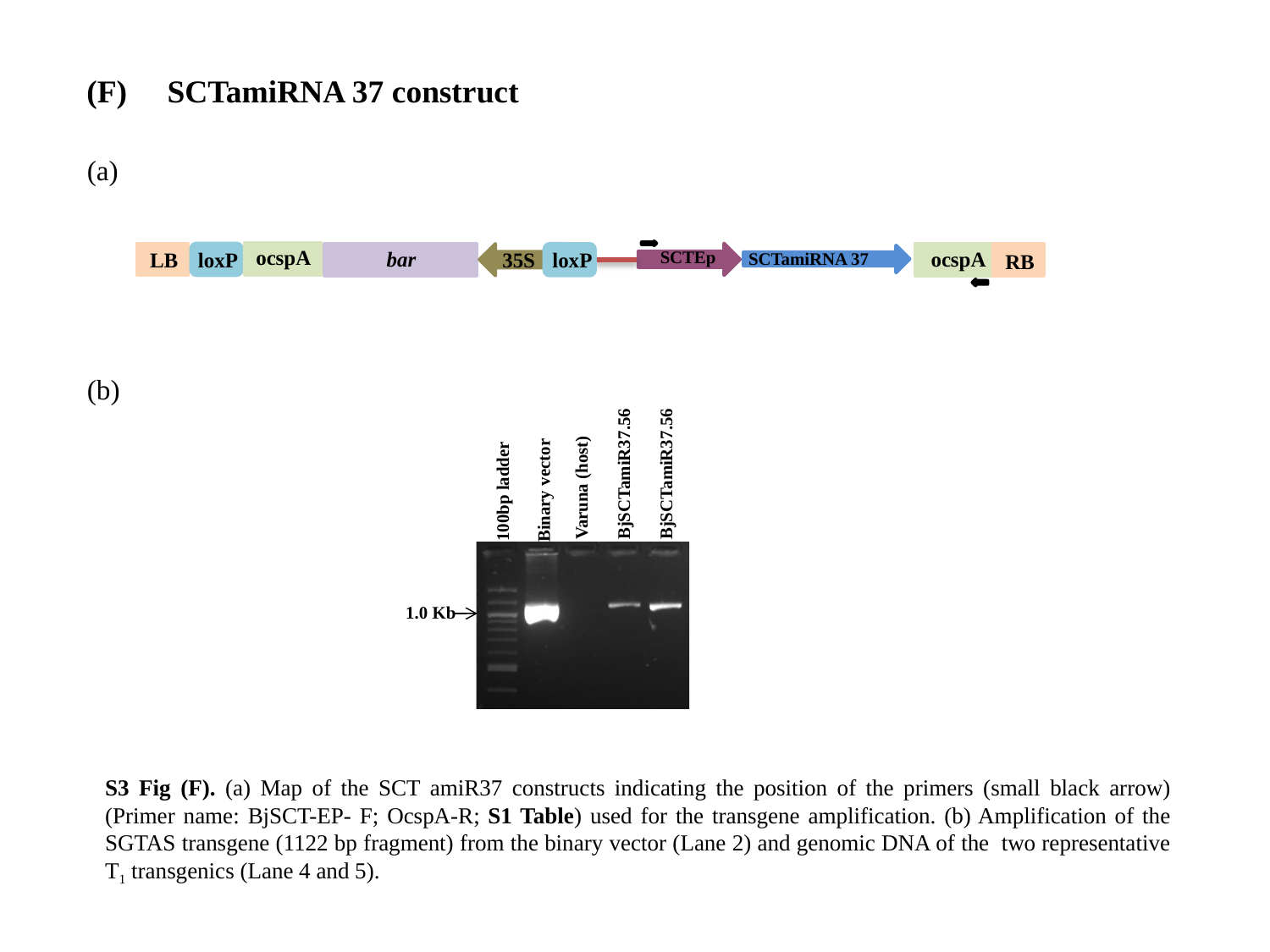

(F) SCTamiRNA 37 construct
(a)
ocspA
bar
SCTEp
loxP
loxP
LB
35S
ocspA
RB
SCTamiRNA 37
(b)
BjSCTamiR37.56
BjSCTamiR37.56
Varuna (host)
Binary vector
100bp ladder
1.0 Kb
S3 Fig (F). (a) Map of the SCT amiR37 constructs indicating the position of the primers (small black arrow) (Primer name: BjSCT-EP- F; OcspA-R; S1 Table) used for the transgene amplification. (b) Amplification of the SGTAS transgene (1122 bp fragment) from the binary vector (Lane 2) and genomic DNA of the two representative T1 transgenics (Lane 4 and 5).
