# Supplementary material for: Development of transgenic Brassica juncea lines for reduced seed sinapine content by perturbing phenylpropanoid pathway genes
Source: PLoS One. 2017 Aug 7;12(8):e0182747. doi: 10.1371/journal.pone.0182747 (PMC5546701; doi:10.1371/journal.pone.0182747)
Supplement: S4 Fig — (PPTX) [file pone.0182747.s004.pptx]

## Slide 1
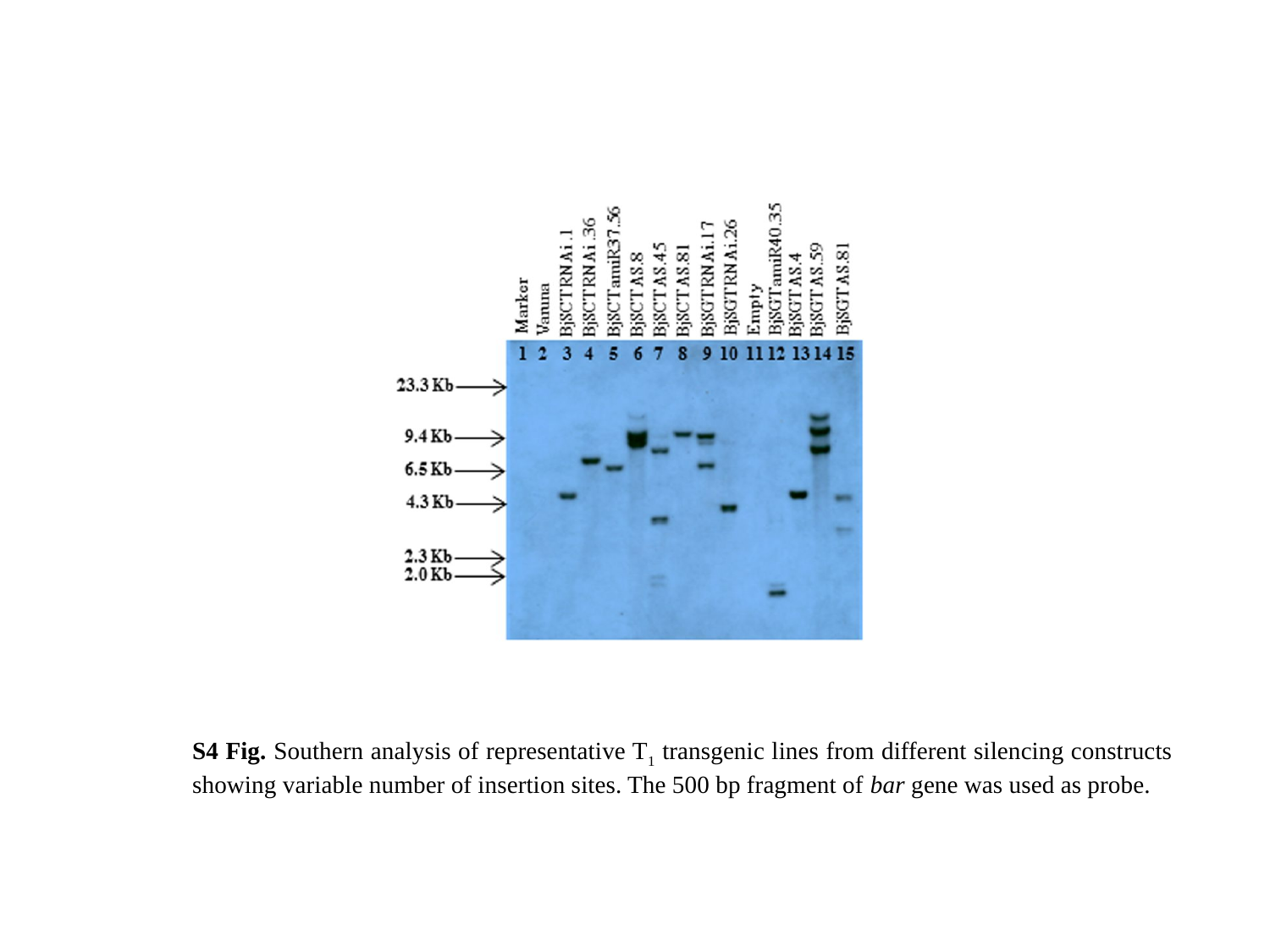

S4 Fig. Southern analysis of representative T1 transgenic lines from different silencing constructs showing variable number of insertion sites. The 500 bp fragment of bar gene was used as probe.
