# Supplementary material for: Development of transgenic Brassica juncea lines for reduced seed sinapine content by perturbing phenylpropanoid pathway genes
Source: PLoS One. 2017 Aug 7;12(8):e0182747. doi: 10.1371/journal.pone.0182747 (PMC5546701; doi:10.1371/journal.pone.0182747)
Supplement: S1 Table — (DOCX) [file pone.0182747.s005.docx]

**S1 Table. Primer sequences of *SGT* and *SCT* genes used in the present study.**

| **Primer Name** | **Primer Sequence 5´-3´** |  |
| --- | --- | --- |
| ***SGT* gene Primers** | |  |
| **Full length gene primer** |  |  |
| BjSGT-IT-F | ATGGAACTATCATCTTCTCCTTTACCT |  |
| BjSGT-IT-R | TTATGACTTTTGCAATAAAAGTTTTTGA |  |
| **Quantitative PCR primers** | |  |
| BjSGTQP-F | ATGTTATGCTTGTATCTTTCCCAGG |  |
| BjSGTQP-R | CGGTCTTGAATCTTGTTGGC |  |
| **Ubiquitin gene primers** |  |  |
| BjUBI-F | TGGGAAGCAGCTCGAGGAT |  |
| BjUBI-R | GACGGTTGACTCTTTCTGGAT |  |
| **Napin Promoter Primers** |  |  |
| BjNap-EP-F | ATTGCGGCCGCCTGCAGTCTCATCCCCTTTTAAACCAA |  |
| BjNap-AB-R | ATTACCGGTAGGATCCGTGTATGTTTTTAATCTTGTTTGTATTG |  |
| **SGT RNAi Primers** | |  |
| Sense strand |  |  |
| BjSGT-SS-F | ATTTCCATGGATGGAACTATCATCTTCTCTCCTTTA |  |
| BjSGT-SS-R | ATTTACCGGTGGCTGTTTCATCACTTCCTTGT |  |
| Antisense Strand |  |  |
| BjSGT-AS-F | ATTTCTGCAGATGGAACTATCATCTTCTCTCCTTTA |  |
| BjSGT-AS-R | ATTTGCTAGCGGCTGTTTCATCACTTCCTTGT |  |
| **SGT Artificial miRNA Primers** | |  |
| BjSGTamiR38-F | TTGCCATGGAAGTGTTTAAAACGCCAAACGCAACGAGAGTTTAGCAG |  |
| BjSGTamiR38-R | TTGGGATCCTGAGTTTAATACGCCAAACGTAAGAAGAGTAAAAGCAA |  |
| BjSGTamiR40-F | TTGCCATGGAAGTAACGACTTTACCTTTCCCTTCGAGAGTTTAGCAG |  |
| BjSGTamiR40-R | TTGGGATCCTGAAACGACATTACCTTTCCTTTGAAGAGTAAAAGCAA |  |
| **SGT antisense primers** |  |  |
| BjSGTAS-F | TCGATGGGATCCTCTAGAATGGAACTATCATCTTCTCCTTTACCT |  |
| BjSGTAS-R | CAGTCACCAGGTTATGACTTTTGCAATAAAAGTTTTTGA |  |
| **Poly A Tail primers** |  |  |
| OcspA-B-F | ATTGGATCCGATCTGTCGACTGCTTTAATGA |  |
| OcspA-P-R | ATTCTGCAGAAGCTTGGTACAATCAGTAAATTGA |  |
|  |  |  |
|  |  |  |
| ***SCT* gene primers** | | |
| **Full length coding sequence primer** | | |
| BjSCT-F | ATGAGAAATCTTTACTTTCTAGTCTTATTTCC | |
| BjSCT-R | TCAGAGAGATTCACCATCAATC | |
| **Quantitative PCR primers** | | |
| BjSCTQP-F | GTCTTATTTCCGTTGAGCATCTTG | |
| BjSCTQP-R | CCAGATTCACCAACACTCACATAC | |
|  |  | |
|  |  | |
|  |  | |
|  |  | |
|  |  | |
|  |  | |
| **Genome Walking Primers** | | |
| BjGW5'SCTPri | CCAGATTCACCAACACTCACATACCCA | |
| BjGW5'SCTSec | AGGAAGATACTTCACATGCAAAGAAGCATCAA | |
| Adaptor primer 1 (AP1)-F | GTAATACGACTCACTATAGGGC | |
| Adaptor primer 2 (AP2)-F | ACTATAGGGCACGCGTGGT | |
| **Endogenous Promoter Primers** | | |
| BjSCT-EP-F | CTACGGCCGCTGCAGCTGCTACGTTTAACTCTT | |
| BjSCT-EP-R | ATTCCATGGAGGATCCCAACCAAAATCAAGATGCTCAAC | |
| **SCT Gene Intron Primers** | | |
| BjSCT-In-F | ATACTGCCATGGATTGCGACCGGTGGTCCTCTTCCTTTTGAGCTC | |
| BjSCT-In-R | ATTGCGGGATCCGCTAGCACATCTCCAGATTCACCAACAC | |
| **SCT RNAi Primers** |  | |
| Sense Strand |  | |
| BjSCT-SS-F | ATACTGCCATGGGTCTTATTTCCGTTGAGCATCTTG | |
| BjSCT-SS-R | ATACTGACCGGTGCATAAGAATATCCAGAACCA | |
| Antisense Strand |  | |
| BjSCT-AS-F | ATACTGGGATCCGTCTTATTTCCGTTGAGCATCTTG | |
| BjSCT-AS-R | ATACTGGCTAGCGCATAAGAATATCCAGAACCA | |
| **MicroRNA Primers** |  | |
| BramiR-F | TTGCTCCATGGAAGTAGGGCTCCTTATAGTTCAAAC | |
| BramiR-R | TTGCTGGATCCTGTAGAGCTCCCTTCAATCC | |
| **Artificial microRNA Primers** | | |
| BjSCTamiR36-F | TTGCCATGGAAGTAATACCTATAAGTAAAGCAGCGAGAGTTTAGCAG | |
| BjSCTamiR36-R | TTGGGATCCTGAAATACCAATAAGTAAAGTAGGAAGAGTAAAAGCAA | |
| BjSCTamiR37-F | TTGCCATGGAAGTTACGTTAAAGAATTTGCCTCCGAGAGTTTAGCAG | |
| BjSCTamiR37-R | TTGGGATCCTGATACGTTTAAGAATTTGCTTCGAAGAGTAAAAGCAA | |
|  |  | |
| **SCT antisense primers** |  | |
| BjSCTAS-F | TCGATGGGATCCTCTAGAATGAGAAATCTTTACTTTCTAGTCTTATTTCC | |
| BjSCTAS-R | CAGTCACCATGGTCAGAGAGATTCACCATCAATC | |
| **Universal primers** |  | |
| T3 Primer-F | AATTAACCCTCACTAAAGGG | |
| T7Primer-R | GTAATACGACTCACTATAGGGC | |
| **Bar gene primers** |  | |
| Bj-bar-F | TCAGATCTCGGTGACGGG | |
| Bj-bar-R | ATGAGCCCAGAACGACGCC | |
| **SGT transgene amplification primers** | | |
| **SGT antisense** |  | |
| BjNap- F | ATTGCGGCCGCCTGCAGTCTCATCCCCTTTTAAACCAA | |
| BjSGTAS-R | CTGTCGTTTTTGTGGGTGAT | |
| **SGT RNAi** |  | |
| Intron-F | ATACTGCCATGGATTGCGACCGGTGGTCCTCTTCCTTTTGAGCTC | |
| OcspA-R | ATTCTGCAGAAGCTTGGTACAATCAGTAAATTGA | |
| **SGT artificial microRNA** |  | |
| BjNap- F | ATTGCGGCCGCCTGCAGTCTCATCCCCTTTTAAACCAA | |
| OcspA-R | ATTCTGCAGAAGCTTGGTACAATCAGTAAATTGA | |
|  |  | |
|  |  | |
| **SCT transgene amplification primers** | | |
| **SCT antisense** |  | |
| BjSCT-EP-F | CTACGGCCGCTGCAGCTGCTACGTTTAACTCTT | |
| OcspA-R | ATTCTGCAGAAGCTTGGTACAATCAGTAAATTGA | |
| **SCT RNAi** |  | |
| BjSCTRNAi-F | CCGTTGAGCATCTTGATTTT | |
| BjSCT-In-R | ATTGCGGGATCCGCTAGCACATCTCCAGATTCACCAACAC | |
| **SCT artificial microRNA** |  | |
| BjSCT-EP-F | CTACGGCCGCTGCAGCTGCTACGTTTAACTCTT | |
| OcspA-R | ATTCTGCAGAAGCTTGGTACAATCAGTAAATTGA | |
