# Supplementary material for: Development of transgenic Brassica juncea lines for reduced seed sinapine content by perturbing phenylpropanoid pathway genes
Source: PLoS One. 2017 Aug 7;12(8):e0182747. doi: 10.1371/journal.pone.0182747 (PMC5546701; doi:10.1371/journal.pone.0182747)
Supplement: S2 Table — (DOCX) [file pone.0182747.s006.docx]

**S2 Table. Sequence identity of *SGT* and *SCT* genes with different *Brassica* species.**

**(A)** Sequence identity (in percentage) of one isolated paralog of *SGT* gene (*BjSGT***)** from *B. juncea* cv. Varuna with paralogs of other *Brassica* species and *Arabidopsis thaliana*

| BjSGT | ---- |  |  |  |  |  |  |  |  |  |  |  |
| --- | --- | --- | --- | --- | --- | --- | --- | --- | --- | --- | --- | --- |
| **BnUGT84A9a** | 98 | ---- |  |  |  |  |  |  |  |  |  |  |
| **BnUGT84A9b** | 99 | 98 | ---- |  |  |  |  |  |  |  |  |  |
| **BnUGT84A9c** | 88 | 89 | 88 | --- |  |  |  |  |  |  |  |  |
| **BnUGT84A9d** | 89 | 89 | 89 | 99 | --- |  |  |  |  |  |  |  |
| **BoUGT84A9a** | 97 | 99 | 98 | 89 | 89 | --- |  |  |  |  |  |  |
| **BoUGT84A9c** | 89 | 89 | 89 | 99 | 98 | 90 | --- |  |  |  |  |  |
| **BrUGT84A9b** | 99 | 97 | 98 | 89 | 90 | 97 | 90 | --- |  |  |  |  |
| **BrUGT84A9d** | 89 | 89 | 89 | 99 | 99 | 89 | 98 | 90 | --- |  |  |  |
| **Bra023872** | 89 | 89 | 89 | 98 | 100 | 89 | 98 | 90 | 99 | --- |  |  |
| **Bra031290** | 99 | 98 | 99 | 88 | 89 | 98 | 89 | 98 | 89 | 89 | --- |  |
| **At3g21560** | 86 | 86 | 85 | 85 | 85 | 86 | 85 | 85 | 85 | 85 | 85 | --- |
|  | **BjSGT** | **BnUGT84A9a** | **BnUGT84A9b** | **BnUGT84A9c** | **BnUGT84A9d** | **BoUGT84A9a** | **BoUGT84A9c** | **BrUGT84A9b** | **BrUGT84A9d** | **Bra023872** | **Bra031290** | **At3g21560** |

Identity was calculated by NCBI blast

**(B)** Sequence identity (in percentage) of two *SCT* CDS variants of *B. juncea* **(*BjSCT1CDS* and *BjSCT2CDS*)** with *SCT* CDS variants of other *Brassica* species and *Arabidopsis* *thaliana*

| **BjSCT1CDS** | ---- |  |  |  |  |  |  |  |  |
| --- | --- | --- | --- | --- | --- | --- | --- | --- | --- |
| **BjSCT2CDS** | 94 | ---- |  |  |  |  |  |  |  |
| **BrSCT(AM706348)** | 99 | 94 | ---- |  |  |  |  |  |  |
| **BoSCT(AM706347)** | 99 | 94 | 99 | ---- |  |  |  |  |  |
| **BnSCT1(AM706349)** | 99 | 94 | 99 | 100 | ---- |  |  |  |  |
| **BnSCT2(AM706350)** | 100 | 94 | 99 | 99 | 99 | ---- |  |  |  |
| **Bra028627** | 91 | 92 | 91 | 91 | 88 | 88 | ---- |  |  |
| **Bra009394** | 88 | 87 | 88 | 88 | 91 | 91 | 85 | ---- |  |
| **At5g09640 (SCT)** | 88 | 88 | 88 | 88 | 88 | 88 | 83 | 85 | ---- |
|  | **BjSCT1CDS** | **BjSCT2CDS** | **BrSCT(AM706348)** | **BoSCT(AM706347)** | **BnSCT1(AM706349)** | **BnSCT2(AM706350)** | **Bra028627** | **Bra009394** | **At5g09640 (SCT)** |

Identity was calculated by NCBI blast
