# Supplementary material for: Development of transgenic Brassica juncea lines for reduced seed sinapine content by perturbing phenylpropanoid pathway genes
Source: PLoS One. 2017 Aug 7;12(8):e0182747. doi: 10.1371/journal.pone.0182747 (PMC5546701; doi:10.1371/journal.pone.0182747)
Supplement: S3 Table — (DOCX) [file pone.0182747.s007.docx]

**S3 Table. Seed sinapine content (mg/g DSW) from T_1_ seeds of transgenics developed from eight suppression constructs of *B. juncea.*** **Lines showing ≤8.19 mg/g DSW (30% less than the value of wt control) are shown in bold**

A. Data of 56 T_1_ seed samples from BjSGTAS construct

| **S. No.** | **Line name** | **Seed Sinapine content**  **(mg/g)** | **S. No.** | **Line name** | **Seed Sinapine content**  **(mg/g)** | **S. No.** | **Line name** | **Seed Sinapine**  **content**  **(mg/g)** |
| --- | --- | --- | --- | --- | --- | --- | --- | --- |
| 1 | **SGTAS.4** | **7.74 ± 0.05** | 21 | SGTAS.42 | 10.31 ± 0.26 | 41 | SGTAS.87 | 11.04 ± 0.07 |
| 2 | **SGTAS.8** | **8.13 ± 0.40** | 22 | SGTAS.43 | 9.59 ± 0.26 | 42 | SGTAS.88 | 10.67 ± 0.34 |
| 3 | SGTAS.9 | 10.53 ± 0.07 | 23 | SGTAS.46 | 12.27 ± 0.04 | 43 | SGTAS.97 | 10.24 ± 0.11 |
| 4 | SGTAS.10 | 10.47 ± 0.03 | 24 | SGTAS.50 | 10.44 ± 0.16 | 44 | SGTAS.98 | 8.42 ± 0.19 |
| 5 | SGTAS.12 | 10.46 ± 0.15 | 25 | **SGTAS.52** | **7.84 ± 0.02** | 45 | SGTAS.100 | 9.52 ± 0.11 |
| 6 | SGTAS.13 | 10.63 ± 0.11 | 26 | SGTAS.54 | 9.31 ± 0.14 | 46 | SGTAS.102 | 9.57 ± 0.12 |
| 7 | SGTAS.14 | 8.43 ± 0.05 | 27 | SGTAS.57 | 12.05 ± 0.62 | 47 | SGTAS.104 | 11.05 ± 0.16 |
| 8 | SGTAS.15 | 10.71 ± 0.24 | 28 | SGTAS.58 | 9.00 ± 0.08 | 48 | SGTAS.105 | 10.10 ± 0.11 |
| 9 | SGTAS.17 | 10.17 ± 0.50 | 29 | **SGTAS.59** | **6.35 ± 0.10** | 49 | SGTAS.109 | 10.11 ± 0.34 |
| 10 | SGTAS.18 | 9.48 ± 0.01 | 30 | SGTAS.62 | 9.34 ± 0.07 | 50 | SGTAS.110 | 8.60 ± 0.10 |
| 11 | SGTAS.24 | 9.65 ± 0.12 | 31 | SGTAS.64 | 10.98 ± 0.30 | 51 | SGTAS.111 | 9.52 ± 0.02 |
| 12 | **SGTAS.25** | **7.87 ± 0.09** | 32 | SGTAS.65 | 11.12 ± 0.07 | 52 | SGTAS.114 | 8.70 ± 0.03 |
| 13 | SGTAS.27 | 10.79 ± 0.30 | 33 | SGTAS.68 | 10.46 ± 0.02 | 53 | SGTAS.116 | 9.36 ± 0.12 |
| 14 | SGTAS.28 | 10.56 ± 0.06 | 34 | SGTAS.69 | 8.73 ± 0.29 | 54 | SGTAS.117 | 10.42 ± 0.69 |
| 15 | SGTAS.29 | 9.55 ± 0.09 | 35 | SGTAS.70 | 8.57 ± 0.86 | 55 | SGTAS.118 | 10.00 ± 0.77 |
| 16 | SGTAS.32 | 10.22 ± 0.12 | 36 | SGTAS.71 | 9.96 ± 0.04 | 56 | SGTAS.123 | 11.88 ± 0.07 |
| 17 | SGTAS.33 | 11.74 ± 0.22 | 37 | SGTAS.73 | 10.72 ± 0.47 | Control | Varuna | 11.70±0.55 |
| 18 | SGTAS.36 | 10.13 ± 0.13 | 38 | SGTAS.76 | 11.26 ± 0.16 |  |  |  |
| 19 | SGTAS.39 | 11.34 ± 0.28 | 39 | **SGTAS.81** | **6.68 ± 0.09** |  |  |  |
| 20 | SGTAS.40 | 10.73 ± 0.29 | 40 | SGTAS.83 | 8.35 ± 0.40 |  |  |  |

B. Data of 43 T_1_ seed samples from BjSGTRNAi construct

| **S. No.** | **Line name** | **Seed Sinapine content (mg/g)** | **S. No.** | **Line name** | **Seed Sinapine content**  **(mg/g)** | **S. No.** | **Line name** | **Seed Sinapine content (mg/g)** |
| --- | --- | --- | --- | --- | --- | --- | --- | --- |
| 1 | **SGTRNAi.1** | **7.77 ± 1.17** | 16 | SGTRNAi.20 | 8.88 ± 0.26 | 31 | **SGTRNAi.38** | **7.2 ± 0.08** |
| 2 | **SGTRNAi.2** | **8.17 ± 0.25** | 17 | SGTRNAi.21 | 8.34 ± 0.21 | 32 | SGTRNAi.40 | 8.66 ± 1.51 |
| 3 | SGTRNAi.4 | 8.25 ± 0.17 | 18 | **SGTRNAi.22** | **6.67 ± 0.03** | 33 | **SGTRNAi.41** | **7.97 ± 0.46** |
| 4 | **SGTRNAi.5** | **8 ± 0.03** | 19 | SGTRNAi.23 | 8.82 ± 0.26 | 34 | **SGTRNAi.42** | **7.73 ± 1.72** |
| 5 | SGTRNAi.7 | 9.24 ± 1.85 | 20 | **SGTRNAi.24** | **6.17 ± 0.5** | 35 | SGTRNAi.43 | 8.29 ± 0.89 |
| 6 | **SGTRNAi.8** | **7.16 ± 0.97** | 21 | **SGTRNAi.25** | **7.77 ± 0.21** | 36 | **SGTRNAi.44** | **7.68 ± 1.76** |
| 7 | SGTRNAi.9 | 8.39 ± 0.76 | 22 | **SGTRNAi.26** | **6.61 ± 0.37** | 37 | **SGTRNAi.45** | **5.66 ± 1.04** |
| 8 | SGTRNAi.10 | 8.22 ± 0.98 | 23 | SGTRNAi.27 | 8.98 ± 0.85 | 38 | SGTRNAi.46 | 8.22 ± 0.86 |
| 9 | **SGTRNAi.11** | **8.05 ± 0.09** | 24 | **SGTRNAi.28** | **7.12 ± 0.93** | 39 | **SGTRNAi.48** | **7.54 ± 1.3** |
| 10 | SGTRNAi.12 | 8.34 ± 0.06 | 25 | SGTRNAi.29 | 8.28 ± 1.11 | 40 | **SGTRNAi.49** | **7.6 ± 0.09** |
| 11 | **SGTRNAi.13** | **7.48 ± 0.43** | 26 | **SGTRNAi.31** | **6.53 ± 1.01** | 41 | **SGTRNAi.50** | **7.57 ± 0.93** |
| 12 | SGTRNAi.14 | 8.26 ± 0.11 | 27 | **SGTRNAi.32** | **8.04 ± 0.7** | 42 | **SGTRNAi.51** | **7.58 ± 0.89** |
| 13 | **SGTRNAi.17** | **7.76 ± 0.65** | 28 | **SGTRNAi.33** | **8.15 ± 0.29** | 43 | **SGTRNAi.53** | **6.49 ± 1.61** |
| 14 | **SGTRNAi.18** | **7.65 ± 0.3** | 29 | **SGTRNAi.34** | **7.12 ± 1.1** | Control | Varuna | 11.70±0.55 |
| 15 | SGTRNAi.19 | 8.67 ± 0.25 | 30 | **SGTRNAi.36** | **7.39 ± 0.18** |  |  |  |

C. Data of 60 T_1_ seed samples from BjSGTamiR38 construct

| **S. No.** | **Line name** | **Seed Sinapine content**  **(mg/g)** | **S. No.** | **Line name** | **Seed Sinapine content**  **(mg/g)** | **S. No.** | **Line name** | **Seed Sinapine content**  **(mg/g)** |
| --- | --- | --- | --- | --- | --- | --- | --- | --- |
| 1 | SGTamiR38.1 | 10.00 ± 0.23 | 22 | SGTamiR38.45 | 12.07 ± 0.05 | 43 | SGTamiR38.86 | 11.34 ± 0.07 |
| 2 | SGTamiR38.2 | 9.36 ± 0.51 | 23 | SGTamiR38.46 | 12.08 ± 0.24 | 44 | SGTamiR38.88 | 11.40 ± 0.15 |
| 3 | SGTamiR38.8 | 11.41 ± 0.39 | 24 | SGTamiR38.48 | 11.08 ± 0.22 | 45 | SGTamiR38.90 | 11.17 ± 0.29 |
| 4 | SGTamiR38.10 | 10.77 ± 0.37 | 25 | SGTamiR38.50 | 10.54 ± 0.10 | 46 | SGTamiR38.92 | 10.64 ± 0.12 |
| 5 | SGTamiR38.12 | 11.65 ± 0.24 | 26 | SGTamiR38.51 | 9.80 ± 0.16 | 47 | SGTamiR38.96 | 12.23 ± 0.18 |
| 6 | SGTamiR38.14 | 11.07 ± 0.28 | 27 | SGTamiR38.56 | 10.86 ± 0.23 | 48 | SGTamiR38.97 | 11.11 ± 0.20 |
| 7 | SGTamiR38.17 | 10.03 ± 0.45 | 28 | SGTamiR38.57 | 10.09 ± 0.09 | 49 | SGTamiR38.99 | 11.94 ± 0.23 |
| 8 | SGTamiR38.19 | 10.84 ± 0.03 | 29 | SGTamiR38.60 | 10.80 ± 0.07 | 50 | SGTamiR38.102 | 10.86 ± 0.22 |
| 9 | SGTamiR38.20 | 10.85 ± 0.02 | 30 | SGTamiR38.61 | 9.17 ± 0.34 | 51 | SGTamiR38.104 | 10.60 ± 0.01 |
| 10 | SGTamiR38.24 | 12.71 ± 0.06 | 31 | SGTamiR38.63 | 10.37 ± 0.61 | 52 | SGTamiR38.110 | 9.84 ± 0.01 |
| 11 | SGTamiR38.25 | 11.45 ± 0.33 | 32 | SGTamiR38.65 | 9.24 ± 0.04 | 53 | SGTamiR38.111 | 11.21 ± 0.11 |
| 12 | SGTamiR38.26 | 12.47 ± 0.92 | 33 | SGTamiR38.67 | 9.90 ± 0.05 | 54 | SGTamiR38.113 | 11.64 ± 0.41 |
| 13 | SGTamiR38.30 | 11.53 ± 0.53 | 34 | SGTamiR38.68 | 9.77 ± 0.10 | 55 | SGTamiR38.124 | 10.59 ± 0.14 |
| 14 | SGTamiR38.31 | 11.47 ± 1.16 | 35 | SGTamiR38.69 | 9.32 ± 0.18 | 56 | SGTamiR38.125 | 9.78 ± 0.24 |
| 15 | SGTamiR38.34 | 12.15 ± 0.48 | 36 | SGTamiR38.71 | 10.72 ± 0.07 | 57 | SGTamiR38.132 | 10.41 ± 0.11 |
| 16 | SGTamiR38.35 | 11.92 ± 0.65 | 37 | SGTamiR38.73 | 12.13 ± 0.23 | 58 | SGTamiR38.135 | 9.80 ± 0.01 |
| 17 | SGTamiR38.36 | 11.27 ± 0.00 | 38 | SGTamiR38.74 | 10.91 ± 0.45 | 59 | SGTamiR38.140 | 10.99 ± 0.64 |
| 18 | SGTamiR38.37 | 13.28 ± 0.22 | 39 | SGTamiR38.77 | 10.66 ± 0.01 | 60 | SGTamiR38.147 | 9.58 ± 0.20 |
| 19 | SGTamiR38.38 | 12.60 ± 0.56 | 40 | SGTamiR38.80 | 11.85 ± 0.19 | Control | Varuna | 11.70±0.55 |
| 20 | SGTamiR38.39 | 13.07 ± 0.52 | 41 | SGTamiR38.81 | 11.88 ± 0.12 |  |  |  |
| 21 | SGTamiR38.40 | 12.76 ± 0.07 | 42 | SGTamiR38.82 | 11.57 ± 0.27 |  |  |  |

D. Data of 135 T_1_ seed samples from BjSGTamiR40 construct

| **S. No.** | **Line name** | **Seed Sinapine content**  **(mg/g)** | **S. No.** | **Line name** | **Seed Sinapine content**  **(mg/g)** | **S. No.** | **Line name** | **Seed Sinapine content**  **(mg/g)** |
| --- | --- | --- | --- | --- | --- | --- | --- | --- |
| 1 | **SGTamiR40.1** | **7.4 ± 0.1** | 46 | SGTamiR40.48 | 9.3 ± 0.25 | 91 | SGTamiR40.98 | 9.98 ± 0.97 |
| 2 | **SGTamiR40.2** | **7.47 ± 0.53** | 47 | **SGTamiR40.49** | **6.42 ± 2.59** | 92 | SGTamiR40.99 | 10.34 ± 0.52 |
| 3 | **SGTamiR40.3** | **7.42 ± 0.67** | 48 | SGTamiR40.50 | 9.56 ± 1.84 | 93 | SGTamiR40.100 | 11.38 ± 0.27 |
| 4 | SGTamiR40.4 | 8.53 ± 0.96 | 49 | SGTamiR40.51 | 9.65 ± 1.44 | 94 | SGTamiR40.101 | 10.08 ± 0.69 |
| 5 | **SGTamiR40.5** | **6.28 ± 0.22** | 50 | **SGTamiR40.52** | **7.77 ± 0.57** | 95 | SGTamiR40.102 | 11.23 ± 0.58 |
| 6 | SGTamiR40.6 | 8.46 ± 0.54 | 51 | **SGTamiR40.54** | **6.73 ± 0.21** | 96 | SGTamiR40.103 | 10.39 ± 1.98 |
| 7 | SGTamiR40.7 | 8.49 ± 0.16 | 52 | SGTamiR40.55 | 8.94 ± 0.03 | 97 | SGTamiR40.105 | 9.88 ± 0.85 |
| 8 | **SGTamiR40.8** | **7.57 ± 1.05** | 53 | SGTamiR40.56 | 10.97 ± 0.59 | 98 | SGTamiR40.106 | 10.23 ± 0.68 |
| 9 | **SGTamiR40.9** | **8.17 ± 0.46** | 54 | SGTamiR40.57 | 9.75 ± 0.26 | 99 | SGTamiR40.107 | 10.1 ± 1.59 |
| 10 | SGTamiR40.10 | 8.71 ± 1.15 | 55 | SGTamiR40.58 | 9.4 ± 1.08 | 100 | SGTamiR40.109 | 9.85 ± 1.56 |
| 11 | SGTamiR40.11 | 10.56 ± 0.8 | 56 | **SGTamiR40.59** | **7.91 ± 0.92** | 101 | SGTamiR40.110 | 10.76 ± 0.49 |
| 12 | **SGTamiR40.12** | **6.18 ± 0.09** | 57 | SGTamiR40.60 | 9.72 ± 0.52 | 102 | SGTamiR40.111 | 9.01 ± 1.39 |
| 13 | SGTamiR40.13 | 8.3 ± 0.92 | 58 | SGTamiR40.61 | 8.47 ± 0.52 | 103 | SGTamiR40.112 | 9.61 ± 1.16 |
| 14 | SGTamiR40.14 | 9.46 ± 1.09 | 59 | SGTamiR40.62 | 10.41 ± 0.77 | 104 | SGTamiR40.116 | 9 ± 0.15 |
| 15 | SGTamiR40.15 | 8.27 ± 1.09 | 60 | SGTamiR40.63 | 10.24 ± 0.47 | 105 | SGTamiR40.117 | 9.56 ± 0.08 |
| 16 | **SGTamiR40.16** | **7.9 ± 0.93** | 61 | SGTamiR40.64 | 9.2 ± 0.26 | 106 | SGTamiR40.119 | 10.71 ± 0.98 |
| 17 | SGTamiR40.17 | 9.55 ± 1.18 | 62 | **SGTamiR40.65** | **6.18 ± 0.25** | 107 | **SGTamiR40.120** | **7.79 ± 0.63** |
| 18 | SGTamiR40.18 | 9.25 ± 0.54 | 63 | SGTamiR40.66 | 8.94 ± 0.99 | 108 | SGTamiR40.122 | 10.53 ± 1.74 |
| 19 | SGTamiR40.19 | 8.31 ± 0.49 | 64 | **SGTamiR40.67** | **7.29 ± 0.92** | 109 | SGTamiR40.131 | 10.67 ± 0.94 |
| 20 | SGTamiR40.20 | 8.96 ± 0.84 | 65 | **SGTamiR40.69** | **7.51 ± 0.13** | 110 | SGTamiR40.132 | 9.57 ± 0.74 |
| 21 | **SGTamiR40.21** | **6.13 ± 0.63** | 66 | SGTamiR40.70 | 10.54 ± 0.74 | 111 | SGTamiR40.133 | 10.27 ± 0.94 |
| 22 | SGTamiR40.22 | 8.75 ± 0.12 | 67 | SGTamiR40.71 | 9.09 ± 0.34 | 112 | SGTamiR40.135 | 10.96 ± 0.29 |
| 23 | SGTamiR40.23 | 9.43 ± 0.55 | 68 | SGTamiR40.72 | 8.99 ± 0.73 | 113 | SGTamiR40.136 | 10.66 ± 0.26 |
| 24 | SGTamiR40.24 | 8.74 ± 0.41 | 69 | SGTamiR40.73 | 9.32 ± 0.75 | 114 | **SGTamiR40.137** | **8.15 ± 0.71** |
| 25 | SGTamiR40.26 | 8.49 ± 0.82 | 70 | SGTamiR40.74 | 9.38 ± 1.34 | 115 | SGTamiR40.138 | 9.74 ± 0.97 |
| 26 | **SGTamiR40.27** | **8.16 ± 0.15** | 71 | **SGTamiR40.75** | **7.86 ± 0.73** | 116 | **SGTamiR40.139** | **8.01 ± 0.98** |
| 27 | **SGTamiR40.28** | **6.25 ± 0.23** | 72 | **SGTamiR40.76** | **5.49 ± 1** | 117 | SGTamiR40.140 | 8.34 ± 0.81 |
| 28 | SGTamiR40.29 | 9.14 ± 0.85 | 73 | **SGTamiR40.77** | **5.32 ± 0.12** | 118 | SGTamiR40.141 | 10.78 ± 0.26 |
| 29 | SGTamiR40.30 | 8.45 ± 0.45 | 74 | SGTamiR40.78 | 9.85 ± 1.55 | 119 | SGTamiR40.143 | 10.67 ± 0.92 |
| **S. No.** | **Line name** | **Seed Sinapine content**  **(mg/g)** | **S. No.** | **Line name** | **Seed Sinapine content**  **(mg/g)** | **S. No.** | **Line name** | **Seed Sinapine content**  **(mg/g)** |
| 30 | SGTamiR40.31 | 8.29 ± 1.1 | 75 | SGTamiR40.79 | 10.15 ± 0.61 | 120 | SGTamiR40.144 | 9.68 ± 0.78 |
| 31 | **SGTamiR40.32** | **7.94 ± 0.64** | 76 | SGTamiR40.80 | 8.99 ± 1.08 | 121 | SGTamiR40.145 | 8.83 ± 0.51 |
| 32 | SGTamiR40.33 | 10.32 ± 0.08 | 77 | **SGTamiR40.81** | **7.13 ± 0.01** | 122 | SGTamiR40.147 | 10.4 ± 0.65 |
| 33 | **SGTamiR40.34** | **8.05 ± 0.63** | 78 | SGTamiR40.82 | 9.84 ± 1.19 | 123 | SGTamiR40.149 | 8.89 ± 0.18 |
| 34 | **SGTamiR40.35** | **6.93 ± 0.01** | 79 | SGTamiR40.83 | 9.97 ± 0.39 | 124 | SGTamiR40.150 | 10.09 ± 0.79 |
| 35 | SGTamiR40.36 | 8.96 ± 1.21 | 80 | SGTamiR40.84 | 8.56 ± 0.35 | 125 | SGTamiR40.151 | 9.14 ± 1.4 |
| 36 | SGTamiR40.38 | 10.04 ± 0.73 | 81 | SGTamiR40.85 | 8.9 ± 0.71 | 126 | SGTamiR40.153 | 9.05 ± 0.49 |
| 37 | SGTamiR40.39 | 8.72 ± 0.57 | 82 | **SGTamiR40.86** | **7.46 ± 0.65** | 127 | SGTamiR40.154 | 10.48 ± 1.72 |
| 38 | SGTamiR40.40 | 9.03 ± 0.75 | 83 | **SGTamiR40.87** | **7.75 ± 1.1** | 128 | SGTamiR40.156 | 10.17 ± 0.01 |
| 39 | **SGTamiR40.41** | **6.99 ± 1.08** | 84 | **SGTamiR40.89** | **7.84 ± 0.86** | 129 | SGTamiR40.157 | 9.62 ± 0.41 |
| 40 | **SGTamiR40.42** | **6.48 ± 0** | 85 | SGTamiR40.92 | 10.91 ± 0.89 | 130 | SGTamiR40.158 | 11.06 ± 0.32 |
| 41 | SGTamiR40.43 | 9.25 ± 0.22 | 86 | SGTamiR40.93 | 9.2 ± 0.85 | 131 | SGTamiR40.159 | 10.93 ± 0.19 |
| 42 | SGTamiR40.44 | 9.79 ± 0.31 | 87 | SGTamiR40.94 | 9.57 ± 0.12 | 132 | SGTamiR40.160 | 10.73 ± 0.99 |
| 43 | SGTamiR40.45 | 11.18 ± 0.17 | 88 | SGTamiR40.95 | 11.18 ± 0.77 | 133 | SGTamiR40.161 | 9.36 ± 0.4 |
| 44 | **SGTamiR40.46** | **7.36 ± 2.03** | 89 | SGTamiR40.96 | 10.52 ± 0.72 | 134 | SGTamiR40.164 | 9.35 ± 0.08 |
| 45 | SGTamiR40.47 | 10.18 ± 0.63 | 90 | SGTamiR40.97 | 10.14 ± 0.26 | 135 | SGTamiR40.165 | 9.35±0.57 |
|  |  |  |  |  |  | Control | Varuna | 11.70±0.55 |

| **S. No.** | **Line name** | **Seed Sinapine content**  **(mg/g)** | **S. No.** | **Line name** | **Seed Sinapine content**  **(mg/g)** | **S. No.** | **Line name** | | **Seed Sinapine content**  **(mg/g)** |
| --- | --- | --- | --- | --- | --- | --- | --- | --- | --- |
| 1 | SCTAS.4 | 9.22 ± 0.58 | 28 | **SCTAS.36** | **7.95 ± 0.82** | 55 | SCTAS.71 | | 9.17 ± 0.03 |
| 2 | SCTAS.5 | 8.93 ± 0.58 | 29 | SCTAS.37 | 9.16 ± 0.51 | 56 | SCTAS.72 | | 8.99 ± 0.58 |
| 3 | **SCTAS.8** | **7.88 ± 1.35** | 30 | SCTAS.39 | 8.55 ± 1.41 | 57 | SCTAS.73 | | 8.53 ± 1.99 |
| 4 | SCTAS.10 | 9.83 ± 0.69 | 31 | SCTAS.40 | 9.27 ± 0.34 | 58 | SCTAS.74 | | 9.06 ± 0.49 |
| 5 | SCTAS.11 | 9.03 ± 0.7 | 32 | SCTAS.41 | 8.34 ± 0.88 | 59 | SCTAS.75 | | 9.04 ± 2.87 |
| 6 | SCTAS.12 | 10.68 ± 0.69 | 33 | SCTAS.42 | 9.62 ± 1.77 | 60 | SCTAS.77 | | 8.33 ± 0.3 |
| 7 | SCTAS.13 | 9.06 ± 0.95 | 34 | SCTAS.43 | 9.13 ± 0.48 | 61 | SCTAS.78 | | 8.23 ± 0.62 |
| 8 | SCTAS.14 | 9.33 ± 1.03 | 35 | **SCTAS.45** | **8.06 ± 0.55** | 62 | **SCTAS.79** | | **8.16 ± 0.57** |
| 9 | SCTAS.15 | 9.07 ± 0.93 | 36 | SCTAS.46 | 9.13 ± 0.28 | 63 | SCTAS.80 | | 8.42 ± 0.53 |
| 10 | SCTAS.16 | 8.49 ± 1.26 | 37 | SCTAS.47 | 9.14 ± 0.31 | 64 | **SCTAS.81** | | **7.9 ± 0.58** |
| 11 | SCTAS.17 | 11.25 ± 1.76 | 38 | SCTAS.48 | 8.91 ± 0.47 | 65 | SCTAS.82 | | 8.58 ± 1.83 |
| 12 | SCTAS.18 | 8.97 ± 1.88 | 39 | SCTAS.49 | 8.52 ± 0.8 | 66 | SCTAS.83 | | 8.73 ± 0.22 |
| 13 | SCTAS.19 | 8.85 ± 0.75 | 40 | SCTAS.50 | 10.16 ± 0.7 | 67 | SCTAS.84 | | 9 ± 0.67 |
| 14 | SCTAS.20 | 11.15 ± 0.79 | 41 | SCTAS.52 | 9.08 ± 1.32 | 68 | SCTAS.85 | | 8.45 ± 0.86 |
| 15 | SCTAS.21 | 9.16 ± 0.6 | 42 | SCTAS.53 | 9.04 ± 0.9 | 69 | SCTAS.87 | | 9.32 ± 1.97 |
| 16 | SCTAS.22 | 9.49 ± 0.87 | 43 | SCTAS.55 | 9.8 ± 0.35 | 70 | SCTAS.142 | | 9.25 ± 0.57 |
| 17 | **SCTAS.23** | **8.15 ± 0.81** | 44 | SCTAS.56 | 8.21 ± 0.48 | 71 | SCTAS.143 | | 9.51 ± 0.51 |
| 18 | SCTAS.24 | 9.99 ± 0.72 | 45 | SCTAS.57 | 9.33 ± 1.18 | 72 | SCTAS.144 | | 9.65 ± 0.98 |
| 19 | SCTAS.25 | 9.45 ± 0.78 | 46 | SCTAS.58 | 8.58 ± 0.65 | 73 | SCTAS.145 | | 10.85 ± 0.59 |
| 20 | SCTAS.27 | 9.45 ± 1.53 | 47 | SCTAS.59 | 8.98 ± 0.17 | 74 | SCTAS.146 | | 8.48 ± 1.65 |
| 21 | SCTAS.28 | 9.33 ± 0.73 | 48 | SCTAS.62 | 8.91 ± 1.77 | 75 | SCTAS.147 | | 8.66 ± 0.09 |
| 22 | SCTAS.30 | 9.67 ± 0.97 | 49 | SCTAS.63 | 8.99 ± 0.52 | 76 | SCTAS.149 | | 9.95 ± 0.9 |
| 23 | SCTAS.31 | 9.65 ± 0.95 | 50 | SCTAS.64 | 8.62 ± 1.62 | 77 | SCTAS.155 | | 9.31 ± 0.63 |
| 24 | SCTAS.32 | 8.43 ± 0.76 | 51 | **SCTAS.65** | **8.19 ± 0.74** | 78 | SCTAS.156 | | 9.45 ± 0.6 |
| 25 | SCTAS.33 | 9.67 ± 0.78 | 52 | SCTAS.66 | 8.41 ± 1.45 | 79 | **SCTAS.157** | | **7.18 ± 0.34** |
| 26 | SCTAS.34 | 8.8 ± 2.88 | 53 | SCTAS.67 | 9.34 ± 1.8 | Control | | Varuna | 11.70±0.55 |
| 27 | SCTAS.35 | 8.91 ± 0.44 | 54 | SCTAS.69 | 8.62 ± 0.66 |  |  | |  |

E. Data of 79 T_1_ seed samples from BjSCTAS construct

F. Data of 33 T_1_ seed samples from BjSCTRNAi construct

| **S. No.** | **Event name** | **Sinapine concentration (mg/g)** | **S. No.** | **Event name** | **Sinapine concentration (mg/g)** |
| --- | --- | --- | --- | --- | --- |
| 1 | **SCTRNAi. 1** | **4.47 ± 0.09** | 18 | SCTRNAi.27 | 9.20 ± 0.08 |
| 2 | SCTRNAi. 4 | 9.53 ± 0.57 | 19 | SCTRNAi.28 | 9.28 ± 0.21 |
| 3 | SCTRNAi. 6 | 9.81 ± 0.49 | 20 | SCTRNAi.29 | 9.00 ± 0.41 |
| 4 | SCTRNAi. 9 | 9.28 ± 0.07 | 21 | SCTRNAi.30 | 9.99 ± 0.34 |
| 5 | SCTRNAi. 10 | 10.98 ± 0.27 | 22 | **SCTRNAi.36** | **7.40 ± 0.16** |
| 6 | SCTRNAi. 11 | 9.68 ± 0.09 | 23 | SCTRNAi.37 | 8.31 ± 0.17 |
| 7 | SCTRNAi. 12 | 9.58 ± 0.06 | 24 | SCTRNAi.38 | 10.36 ± 0.41 |
| 8 | SCTRNAi. 13 | 9.87 ± 0.31 | 25 | SCTRNAi.39 | 8.86 ± 0.19 |
| 9 | SCTRNAi.14 | 9.68 ± 0.12 | 26 | SCTRNAi.43 | 9.08 ± 0.23 |
| 10 | SCTRNAi.16 | 9.76 ± 0.15 | 27 | SCTRNAi.49 | 10.26 ± 1.06 |
| 11 | SCTRNAi.17 | 9.42 ± 0.14 | 28 | **SCTRNAi.52** | **7.72 ± 0.67** |
| 12 | SCTRNAi.18 | 8.54 ± 0.11 | 29 | SCTRNAi.53 | 9.28 ± 0.40 |
| 13 | SCTRNAi.20 | 9.65 ± 0.28 | 30 | SCTRNAi.55 | 9.48 ± 0.04 |
| 14 | SCTRNAi.22 | 9.86 ± 0.32 | 31 | SCTRNAi.56 | 8.68 ± 0.25 |
| 15 | SCTRNAi.23 | 10.19 ± 0.26 | 32 | SCTRNAi.58 | 9.30 ± 0.08 |
| 16 | SCTRNAi.24 | 9.30 ± 0.10 | 33 | **SCTRNAi.60** | **7.88 ± 0.04** |
| 17 | **SCTRNAi.25** | **8.10 ± 0.31** | Control | Varuna | 11.70±0.55 |

G. Data of 56 T_1_ seed samples from BjSCTamiR36 construct

| **S. No.** | **Event name** | **Sinapine concentration (mg/g)** | **S. No.** | **Event name** | **Sinapine concentration (mg/g)** | **S. No.** | **Event name** | **Sinapine concentration (mg/g)** |
| --- | --- | --- | --- | --- | --- | --- | --- | --- |
| 1 | SCTamiR36.55 | 11.67 ± 0.35 | 20 | SCTamiR36.88 | 11.34 ± 0.42 | 39 | SCTamiR36.134 | 10.14 ± 0.31 |
| 2 | SCTamiR36.57 | 10.35 ± 0.18 | 21 | SCTamiR36.90 | 11.56 ± 0.16 | 40 | SCTamiR36.135 | 10.44 ± 0.16 |
| 3 | SCTamiR36.59 | 9.75 ± 0.35 | 22 | SCTamiR36.93 | 10.45 ± 0.06 | 41 | SCTamiR36.137 | 10.44 ± 0.23 |
| 4 | SCTamiR36.61 | 12.63 ± 0.06 | 23 | SCTamiR36.94 | 11.48 ± 0.10 | 42 | SCTamiR36.139 | 10.25 ± 0.18 |
| 5 | SCTamiR36.65 | 10.85 ± 1.26 | 24 | SCTamiR36.97 | 8.62 ± 0.53 | 43 | SCTamiR36.140 | 11.71 ± 0.58 |
| 6 | SCTamiR36.67 | 11.86 ± 0.11 | 25 | SCTamiR36.99 | 12.01 ± 0.08 | 44 | SCTamiR36.141 | 10.72 ± 0.23 |
| 7 | SCTamiR36.68 | 10.47 ± 0.55 | 26 | SCTamiR36.100 | 10.01 ± 0.06 | 45 | SCTamiR36.144 | 10.38 ± 0.08 |
| 8 | SCTamiR36.69 | 11.86 ± 0.46 | 27 | SCTamiR36.101 | 11.26 ± 0.05 | 46 | SCTamiR36.158 | 10.95 ± 0.28 |
| 9 | SCTamiR36.72 | 11.28 ± 0.01 | 28 | SCTamiR36.103 | 11.27 ± 0.22 | 47 | SCTamiR36.159 | 9.91 ± 0.36 |
| 10 | SCTamiR36.73 | 11.85 ± 0.07 | 29 | SCTamiR36.105 | 10.81 ± 0.1 | 48 | SCTamiR36.160 | 9.94 ± 0.22 |
| 11 | SCTamiR36.74 | 9.93 ± 0.03 | 30 | SCTamiR36.106 | 9.61 ± 0.38 | 49 | SCTamiR36.161 | 9.85 ± 0.14 |
| 12 | SCTamiR36.76 | 11.10 ± 0.07 | 31 | SCTamiR36.108 | 10.17 ± 0.07 | 50 | SCTamiR36.164 | 10.24 ± 0.42 |
| 13 | SCTamiR36.79 | 8.71 ± 0.24 | 32 | SCTamiR36.109 | 11.08 ± 0.62 | 51 | SCTamiR36.165 | 10.48 ± 0.32 |
| 14 | SCTamiR36.81 | 8.63 ± 0.17 | 33 | SCTamiR36.110 | 10.99 ± 0.21 | 52 | SCTamiR36.173 | 10.04 ± 0.21 |
| 15 | SCTamiR36.83 | 9.22 ± 0.12 | 34 | SCTamiR36.111 | 10.27 ± 0.37 | 53 | SCTamiR36.175 | 8.77 ± 0.53 |
| 16 | SCTamiR36.84 | 10.02 ± 0.01 | 35 | SCTamiR36.113 | 10.08 ± 0.38 | 54 | SCTamiR36.179 | 10.22 ± 0.36 |
| 17 | SCTamiR36.85 | 11.07 ± 0.32 | 36 | SCTamiR36.116 | 10.74 ± 0.06 | 55 | SCTamiR36.180 | 10.52 ± 0.2 |
| 18 | SCTamiR36.86 | 11.04 ± 0.07 | 37 | SCTamiR36.117 | 12.31 ± 0.3 | 56 | SCTamiR36.189 | 9.2 ± 0.22 |
| 19 | SCTamiR36.87 | 12.06 ± 0.23 | 38 | SCTamiR36.119 | 9.49 ± 0.2 | Control | Varuna | 11.70±0.55 |

H. Data of 62 T_1_ seed samples from BjSCTamiR37 construct

| **S. No.** | **Event name** | **Sinapine concentration (mg/g)** | **S. No.** | **Event name** | **Sinapine concentration (mg/g)** | **S. No.** | **Event name** | **Sinapine concentration (mg/g)** |
| --- | --- | --- | --- | --- | --- | --- | --- | --- |
| 1 | SCTamiR37.1 | 10.18 ± 0.03 | 22 | SCTamiR37.46 | 10.36 ± 0.34 | 43 | SCTamiR37.84 | 11.23 ± 0.46 |
| 2 | SCTamiR37.5 | 10.91 ± 0.03 | 23 | SCTamiR37.50 | 11.87 ± 0.19 | 44 | SCTamiR37.86 | 9.62 ± 1.13 |
| 3 | SCTamiR37.12 | 11.07 ± 0.36 | 24 | SCTamiR37.52 | 11.37 ± 0.13 | 45 | SCTamiR37.88 | 11.02 ± 0.22 |
| 4 | SCTamiR37.14 | 10.05 ± 0.87 | 25 | SCTamiR37.53 | 10.26 ± 0.08 | 46 | SCTamiR37.90 | 10.03 ± 0.2 |
| 5 | SCTamiR37.15 | 9.00 ± 0.01 | 26 | SCTamiR37.54 | 11.31 ± 0.15 | 47 | SCTamiR37.91 | 8.62 ± 0.05 |
| 6 | SCTamiR37.18 | 10.35 ± 0.03 | 27 | **SCTamiR37.56** | **7.05 ± 0.05** | 48 | SCTamiR37.92 | 9.97 ± 0.23 |
| 7 | SCTamiR37.19 | 10.22 ± 0.15 | 28 | SCTamiR37.57 | 10.68 ± 1.29 | 49 | SCTamiR37.99 | 9.14 ± 0.59 |
| 8 | SCTamiR37.22 | 10.44 ± 0.13 | 29 | SCTamiR37.58 | 10.79 ± 0.08 | 50 | SCTamiR37.101 | 9.63 ± 0.09 |
| 9 | SCTamiR37.23 | 10.75 ± 0.19 | 30 | SCTamiR37.59 | 10.43 ± 0.28 | 51 | SCTamiR37.105 | 11.04 ± 0.28 |
| 10 | SCTamiR37.24 | 9.77 ± 0.04 | 31 | SCTamiR37.63 | 11.43 ± 0.17 | 52 | SCTamiR37.108 | 9.59 ± 0.05 |
| 11 | SCTamiR37.27 | 10.61 ± 0.57 | 32 | SCTamiR37.66 | 8.26 ± 0.2 | 53 | SCTamiR37.110 | 11.3 ± 0.16 |
| 12 | SCTamiR37.28 | 10.53 ± 0.02 | 33 | SCTamiR37.67 | 10.92 ± 0.04 | 54 | SCTamiR37.112 | 9.26 ± 0.11 |
| 13 | SCTamiR37.31 | 9.13 ± 0.23 | 34 | SCTamiR37.68 | 10.09 ± 0.33 | 55 | SCTamiR37.113 | 10.18 ± 0.13 |
| 14 | SCTamiR37.34 | 11.16 ± 0.07 | 35 | SCTamiR37.71 | 11.25 ± 0.6 | 56 | SCTamiR37.115 | 12.42 ± 0.04 |
| 15 | SCTamiR37.36 | 11.98 ± 0.04 | 36 | SCTamiR37.73 | 8.93 ± 0.07 | 57 | SCTamiR37.120 | 10.39 ± 0.16 |
| 16 | SCTamiR37.40 | 10.11 ± 0.43 | 37 | SCTamiR37.75 | 11.08 ± 0.43 | 58 | SCTamiR37.123 | 9.75 ± 0.16 |
| 17 | SCTamiR37.41 | 11.86 ± 0.36 | 38 | SCTamiR37.76 | 11.49 ± 0.03 | 59 | SCTamiR37.127 | 10.68 ± 0.46 |
| 18 | SCTamiR37.42 | 11.54 ± 0.62 | 39 | SCTamiR37.79 | 9.6 ± 0.44 | 60 | SCTamiR37.129 | 10.74 ± 0.14 |
| 19 | SCTamiR37.43 | 10.15 ± 0.56 | 40 | SCTamiR37.80 | 11.48 ± 0.6 | 61 | SCTamiR37.132 | 10.42 ± 0.51 |
| 20 | SCTamiR37.44 | 10.72 ± 0.29 | 41 | SCTamiR37.81 | 8.76 ± 0.65 | 62 | SCTamiR37.133 | 11.14 ± 0.66 |
| 21 | SCTamiR37.45 | 12.96 ± 0.22 | 42 | SCTamiR37.83 | 10.03 ± 0.1 | Control | Varuna | 11.70±0.55 |
