# Supplementary material for: Development of transgenic Brassica juncea lines for reduced seed sinapine content by perturbing phenylpropanoid pathway genes
Source: PLoS One. 2017 Aug 7;12(8):e0182747. doi: 10.1371/journal.pone.0182747 (PMC5546701; doi:10.1371/journal.pone.0182747)
Supplement: S4 Table — (DOCX) [file pone.0182747.s008.docx]

| **S.No.** | **Name of the Construct** | **Number of lines** | | **Percentage of lines showing**  **≤ 30% sinapine content** | **Name of the lines** |
| --- | --- | --- | --- | --- | --- |
|  |  | **Total analyzed (T_1_ seeds)** | **Lines showing**  **≤ 30% sinapine content** |  |  |
|  | **SGT constructs** |  |  |  |  |
| 1 | BjSGTAS | 56 | 6 | 10.71 | BjSGTAS.4, 8, 25, 52, 59, and 81 |
| 2 | BjSGTRNAi | 43 | 28 | 65.1 | BjSGTRNAi.1, 2, 5, 8, 11, 13, 17, 18, 22, 24, 25, 26, 28, 31, 32, 33,34, 36, 38, 41, 42, 44, 45, 48, 49, 50, 51, and 53 |
| 3 | BjSGTamiR38 | 60 | 0 | 0 |  |
|  | BjSGTamiR40 | 135 | 34 | 25.1 | BjSGTamiR40.1, 2, 3, 5, 8, 9, 12, 16, 21, 27, 28, 32, 34, 35, 41, 42, 46, 49, 52, 54, 59, 65, 67, 69, 75, 76, 77, 81, 86, 87, 89, 120, 137 and 139 |
|  | Total | 294 | 68 (23.12%) |  |  |
|  | **SCT constructs** |  |  |  |  |
| 1 | BjSCTAS | 79 | 8 | 10.12 | BjSCTAS.8, 23, 36, 45, 65, 79, 81, and 157 |
| 2 | BjSCTRNAi | 33 | 5 | 15.15 | BjSCTRNAi.1, 25, 36, 52, and 60 |
| 3 | BjSCTamiR36 | 56 | 0 | 0 |  |
| 4 | BjSCTamiR37 | 62 | 1 | 1.6 | BjSCTamiR37.56 |
|  | Total | 230 | 14 (6.08%) |  |  |
|  | Varuna (Control) |  | 11.70 ± 0.55 |  |  |

**S4 Table. T_1_ lines showing ≥30% reduction in seed sinapine content than the wild type genotype (Varuna).**

Figure in parentheses are in percentage from total
