# Supplementary material for: Development of transgenic Brassica juncea lines for reduced seed sinapine content by perturbing phenylpropanoid pathway genes
Source: PLoS One. 2017 Aug 7;12(8):e0182747. doi: 10.1371/journal.pone.0182747 (PMC5546701; doi:10.1371/journal.pone.0182747)
Supplement: S1 Appendix — (DOCX) [file pone.0182747.s009.docx]

**S1 Appendix: Development of different transformation constructs for *SGT* and *SCT* genes.**

For the development of different silencing constructs a modified binary vector (pPZP200lox) containing *bar* gene (confer basta resistance for selection) cloned within *lox* P sites, was used (Arumugam et al. 2007). All the eight constructs were assembled in intermediate pBSK+ vector (ampicillin selection), first of all terminator sequence of octopine synthase gene was amplified and introduced as a 5´-end *Bam*HI and 3´-end *Pst*I restriction fragment in pBSK+ vector. A 750 bp fragment of *SCT* gene upstream region (used as promoter) was amplified with forward and reverse primers contained *Eag*I-*Pst*I and *Nco*I-*Bam*HI sites respectively. Promoter fragment was cloned with 5´-end *Eag*I and 3´-end *Bam*HI fragment, it results in a pBSK+::SGTnap/SCTep-MCS-ocspA vector backbone. For *SGT* gene constructs similar procedure was followed except napin gene promoter was used in place of native promoter, it was amplified from the pCGMCP22 vector (available in the lab) with forward and reverse primers with *Eag*I/*Pst*I and *Bam*HI/*Age*I restriction endonuclease sites, respectively.

**Antisense RNA constructs**

For antisense RNA constructs development for both genes, full length coding sequences (1494 bp for *SGT* and 1410 bp for *SCT* genes) were used in reverse orientations. These coding sequences were amplified with primers having *Bam*HI and *Nco*I sites and cloned in pBSK+:: SGTnap/SCTep-MCS-OcspA, it results in pBSK+:: SGTnap/SCTep-*SGT*/*SCT*AS-OcspA (Figure a and b shown below).

1. (b)

The map of binary vector pPZP200 lox SGTAS (a) and pPZP200 lox SCTAS (b), revealed the positions of SGT and SCT antisense cassette (Pnap-SGTAS-ocspA and Pscte-SCTAS-ocspA), lox-P, bar cassette (ocspA-bar-35S), LB and RB. The sites *Bam*HI, *Kpn*I and *Pst*I used for restriction analysis of binary vector have been marked

**RNAi constructs**

For RNAi, a 164 bp spacer sequence (*SCT* gene I intron) was amplified with primers having *Nco*I-*Age*I and *Nhe*I-*Bam*HI restriction sites and introduced as 5´-end *Nco*I and 3´-end *Bam*HI fragment between promoter and terminator sequence in said pBSK+ backbone. A 359 and 388bp consensus sequence of *SGT* (**S2 appendix**)and *SCT* (**S3 appendix**) genes were amplified using sequence specific primers and cloned in sense and antisense orientation flanking the intron sequence. The sense fragment as *Nco*I/*Age*I and antisense as *Nhe*I/*Bam*HI fragments were cloned in pBSK+, it results in pBSK+:: SGTnap/SCTep-SS-IN-AS-OcspA cassette (Figure c and d shown below).

(c) (d)

The map of binary vector pPZP200 lox SGTRNAi (a) and pPZP200 lox SCTRNAi (b) showing the positions of SGT and SCT hairpin cassette (Pnap-SS-IN-AS-ocspA and Pscte-SS-IN-AS-ocspA), lox-P sites, bar cassette (ocspA-bar-35S), LB and RB. The restrictions sites for *Bam*HI, *Nco*I & *Kpn*I, *Nhe*I & *Xba*I and *Pst*I used for restriction analysis of binary vector have also been marked

**Artificial microRNA constructs**

The artificial micro RNA constructs were developed in the same pBSK+:: SGTnap/SCTep-MCS-OcspA vector backbone. A 21mer artificial microRNA was designed from web microRNA designer (WMD) tool and introduced into Bra miR159a microRNA backbone using PCR technique. The primers were designed having in built 21mer artificial microRNAs and *Nco*I/*Bam*HI restriction sites, PCR was performed using Bra miR159a microRNA precursor as template and it replaced the miR159a microRNA with *SGT* and *SCT* genes artificial microRNAs. These *SGT* and *SCT* genes artificial microRNAs precursor were cloned as 5´-end *Nco*I and 3´-end *Bam*HI fragment in pBSK+:: SGTnap/SCTep-MCS-OcspA, it results in pBSK+:: SGTnap/SCTep-*SGT*/*SCT*amiRNA-OcspA (Figure e and f shown below).

(e) (f)

The map of binary vector pPZP200 lox SGTamiR38/40 and pPZP200 lox SCTamiR36/37, revealed the positions of SGTamiR38/40 and SCTamiR36/37 cassette (Pnap-SGTamiR38/40-ocspA and Pscte-SCTamiR36/37-ocspA), lox-P, bar cassette (ocspA-bar-35S), LB and RB. The sites *Bam*HI & *Not*I, *Nco*I & *Kpn*I, and *Pst*I used for restriction analysis of binary vector have been marked

All cassettes of different silencing constructs, were excised from pBSK+ intermediate vector as *Pst*I fragments and cloned in similarly digested pPZP200lox binary vector, it results in pPZP200:35Sde-bar-ocspA:: SGTnap/SCTep-cassettes-OcspA transformation constructs. Transformation vectors were mobilized into *A. tumefaciens* strain GV3101 using electroporation method. All the manipulations were performed using standard protocols, primers used in the present study are provided in Online Resource 1.
