# Supplementary material for: Development of transgenic Brassica juncea lines for reduced seed sinapine content by perturbing phenylpropanoid pathway genes
Source: PLoS One. 2017 Aug 7;12(8):e0182747. doi: 10.1371/journal.pone.0182747 (PMC5546701; doi:10.1371/journal.pone.0182747)
Supplement: S2 Appendix — (PPTX) [file pone.0182747.s010.pptx]

## Slide 1
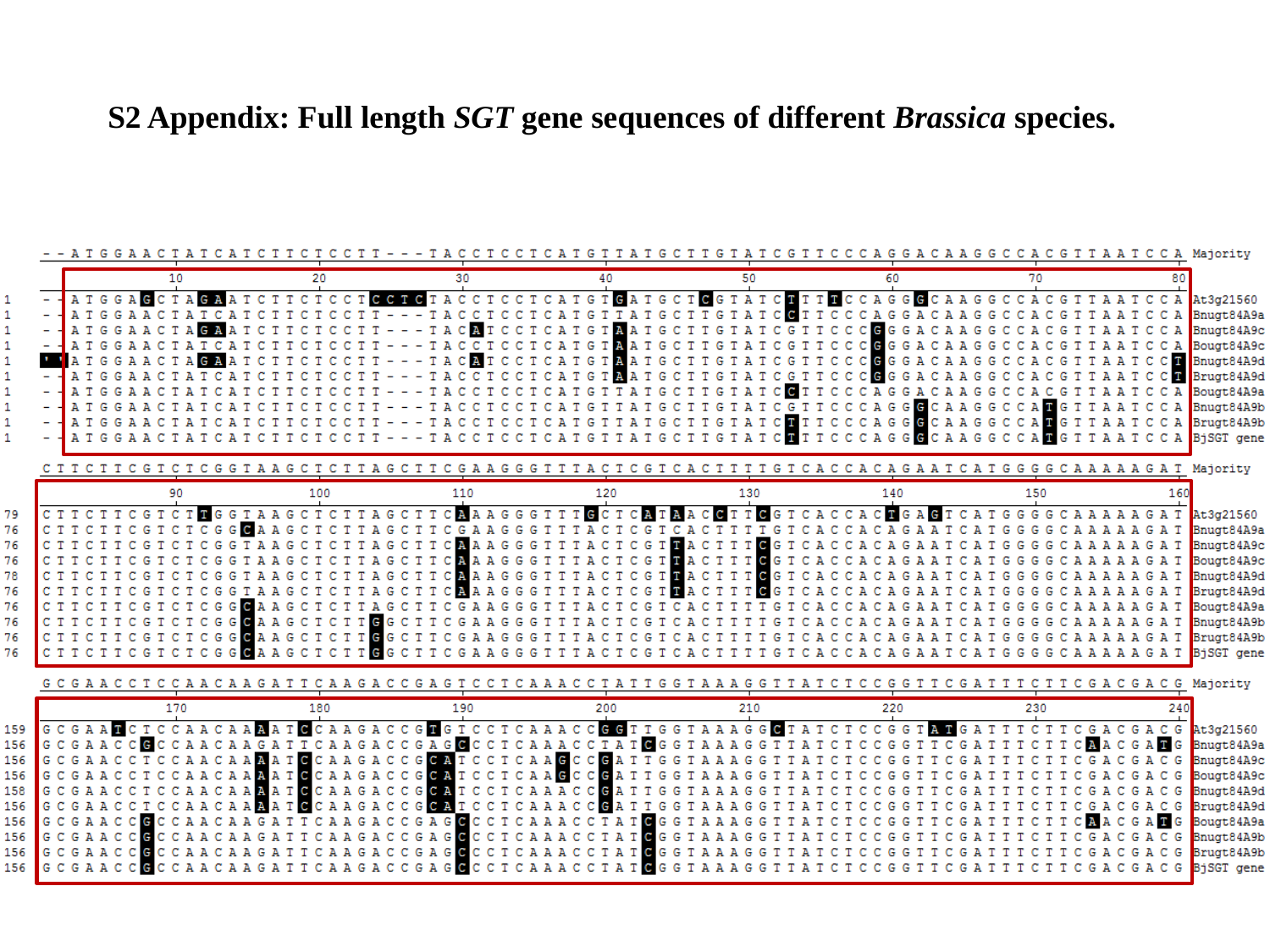

S2 Appendix: Full length SGT gene sequences of different Brassica species.

## Slide 2
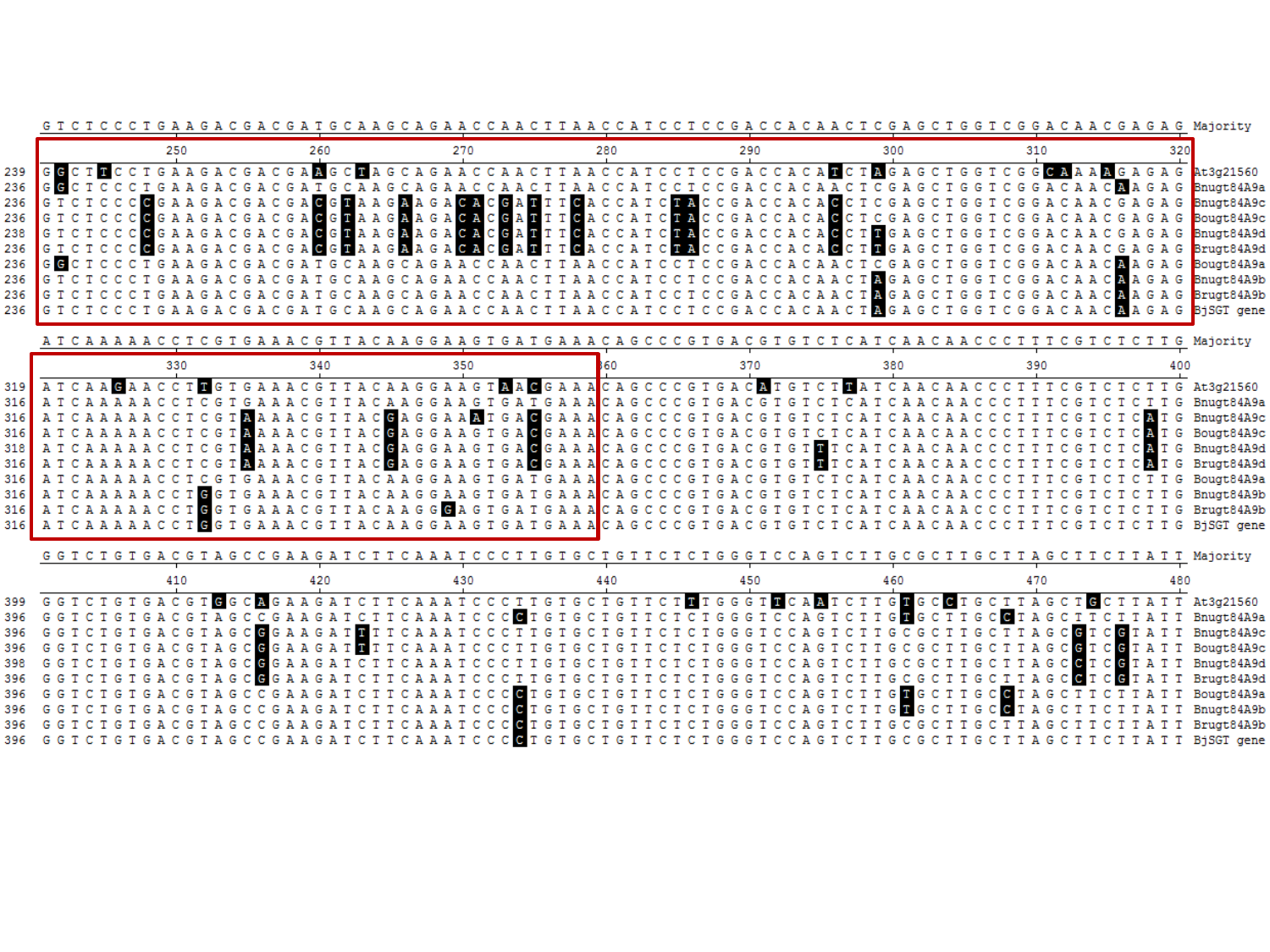

## Slide 3
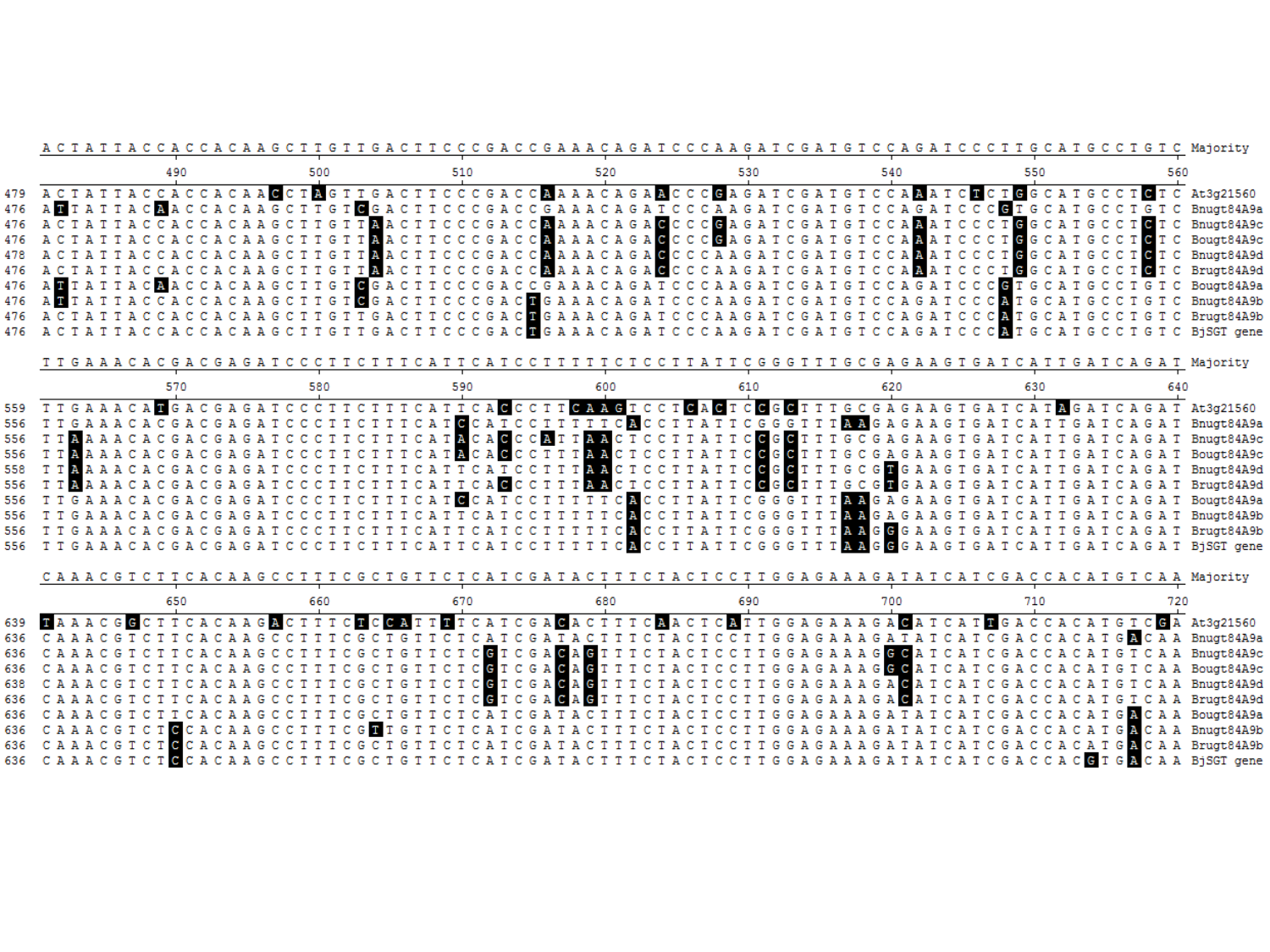

## Slide 4
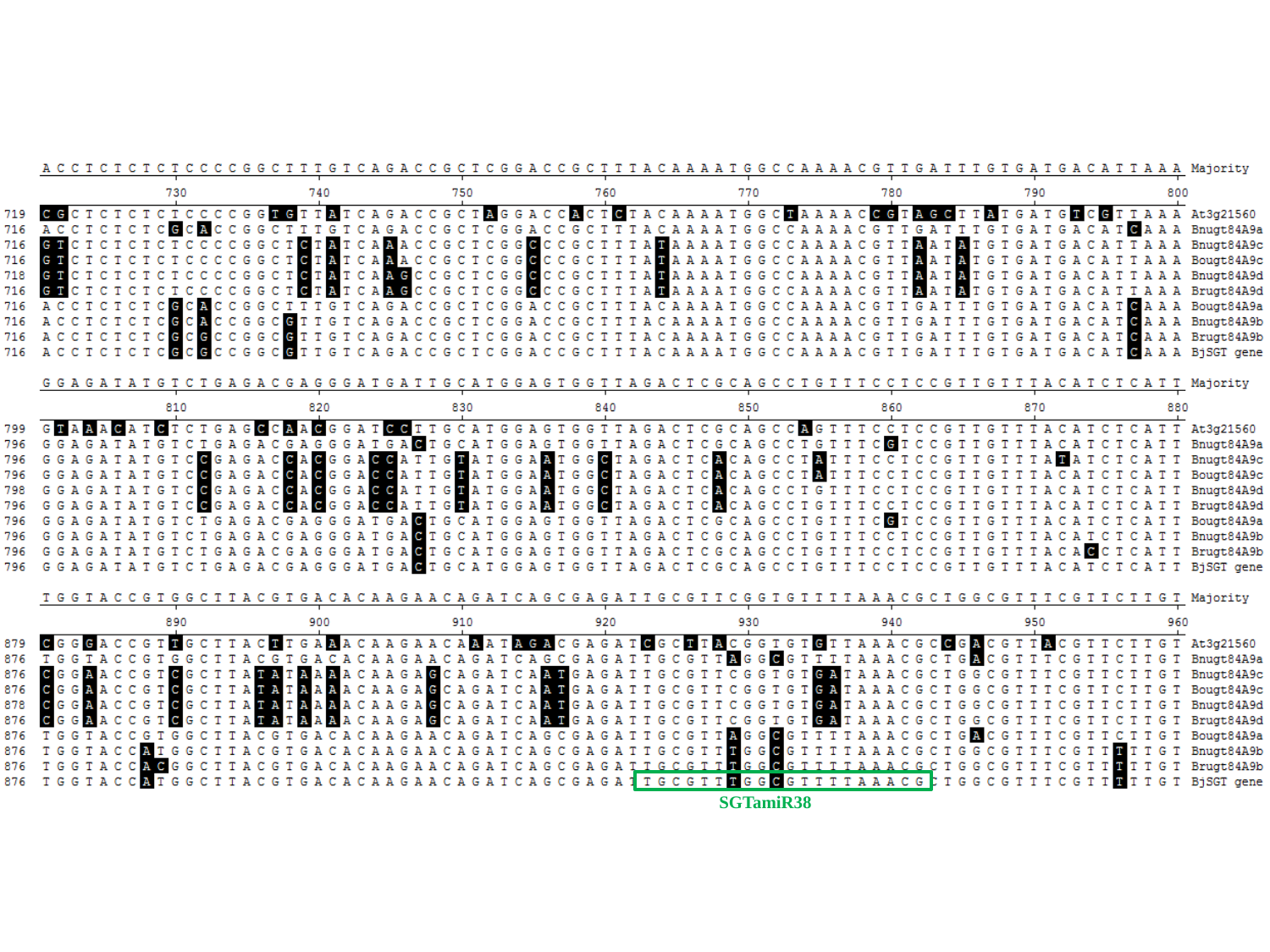

SGTamiR38

## Slide 5
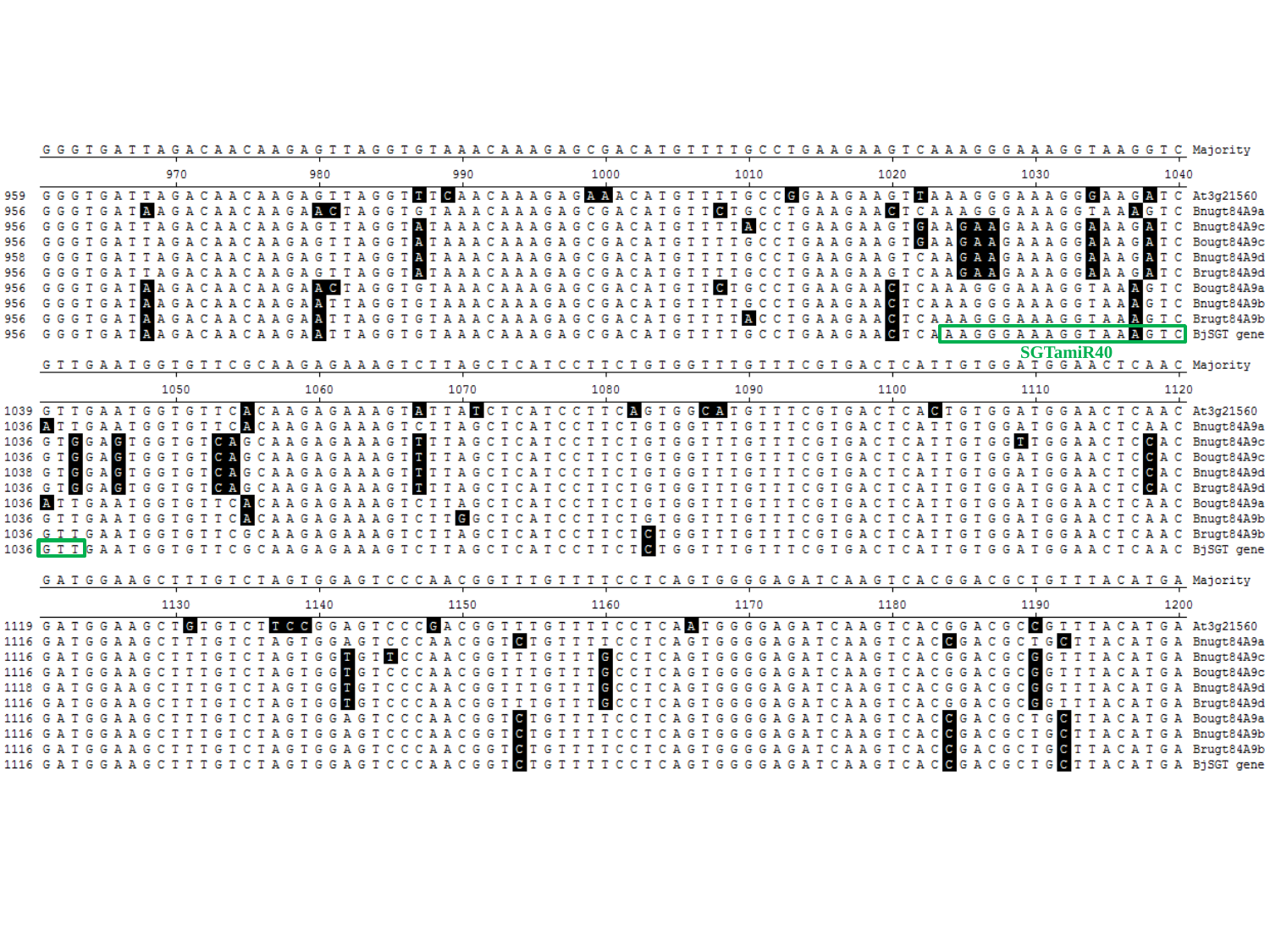

SGTamiR40

## Slide 6
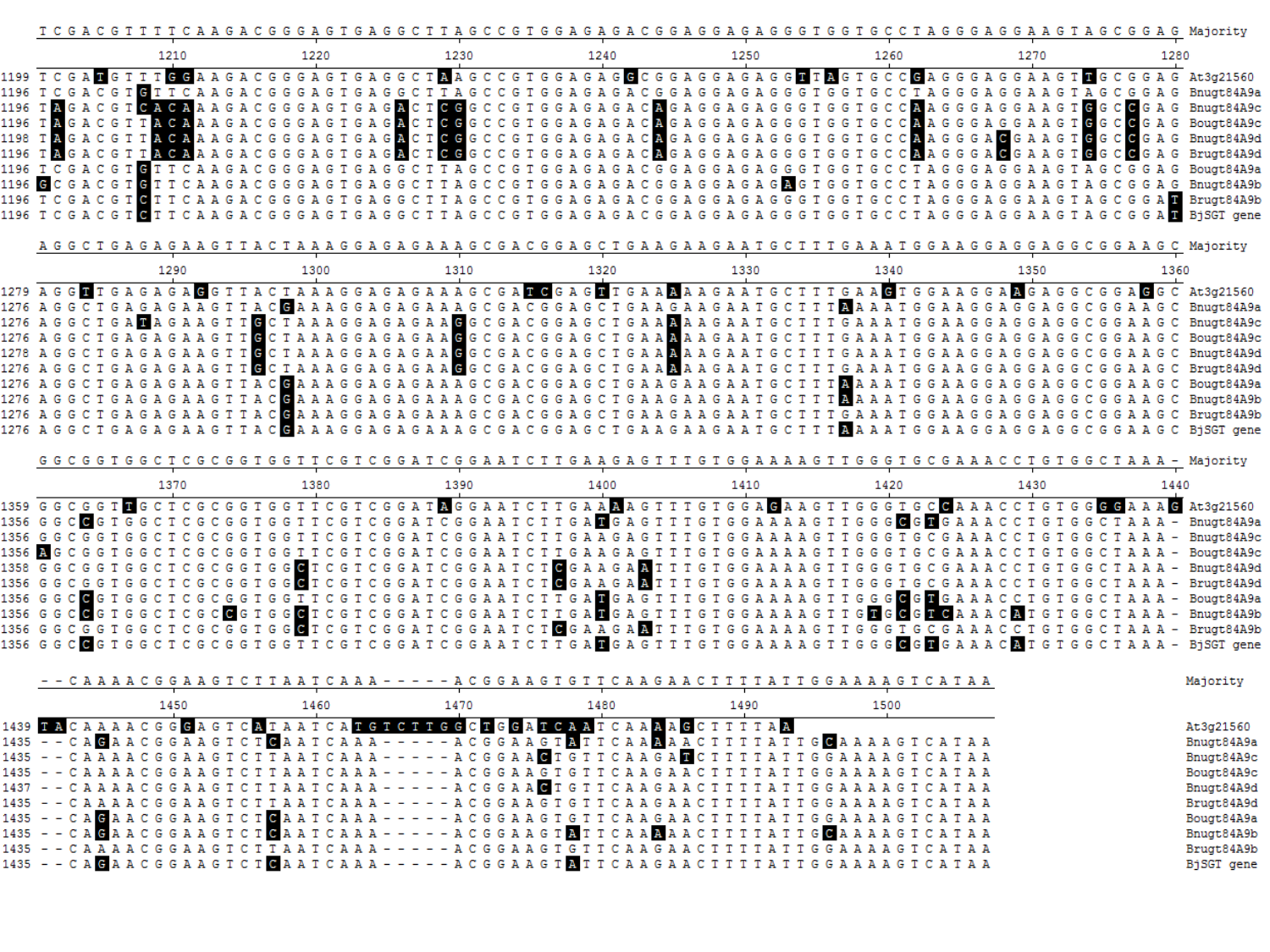

## Slide 7
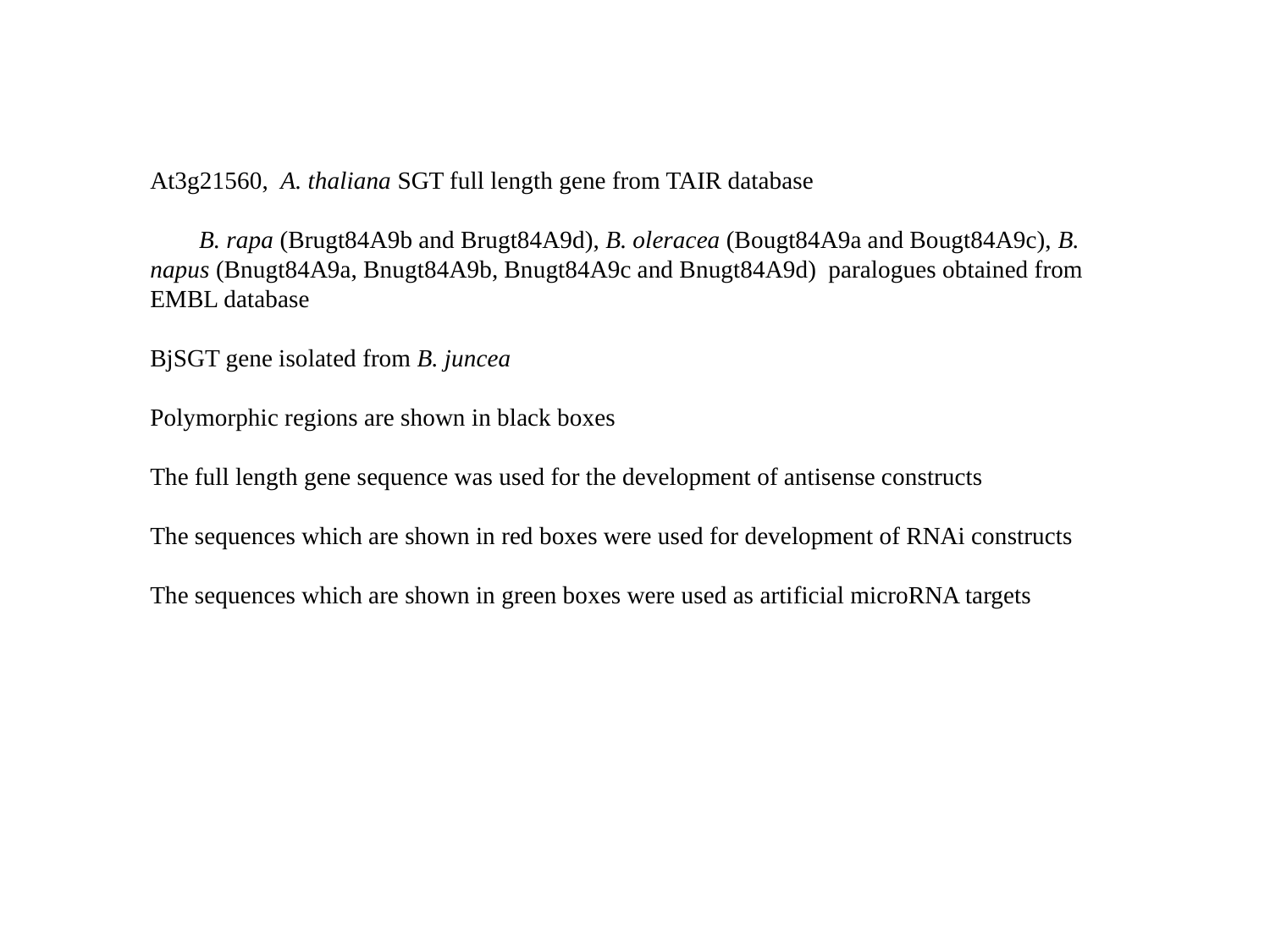

At3g21560, A. thaliana SGT full length gene from TAIR database
 B. rapa (Brugt84A9b and Brugt84A9d), B. oleracea (Bougt84A9a and Bougt84A9c), B. napus (Bnugt84A9a, Bnugt84A9b, Bnugt84A9c and Bnugt84A9d) paralogues obtained from EMBL database
BjSGT gene isolated from B. juncea
Polymorphic regions are shown in black boxes
The full length gene sequence was used for the development of antisense constructs
The sequences which are shown in red boxes were used for development of RNAi constructs
The sequences which are shown in green boxes were used as artificial microRNA targets
