# Supplementary material for: Development of transgenic Brassica juncea lines for reduced seed sinapine content by perturbing phenylpropanoid pathway genes
Source: PLoS One. 2017 Aug 7;12(8):e0182747. doi: 10.1371/journal.pone.0182747 (PMC5546701; doi:10.1371/journal.pone.0182747)
Supplement: S3 Appendix — (PPTX) [file pone.0182747.s011.pptx]

## Slide 1
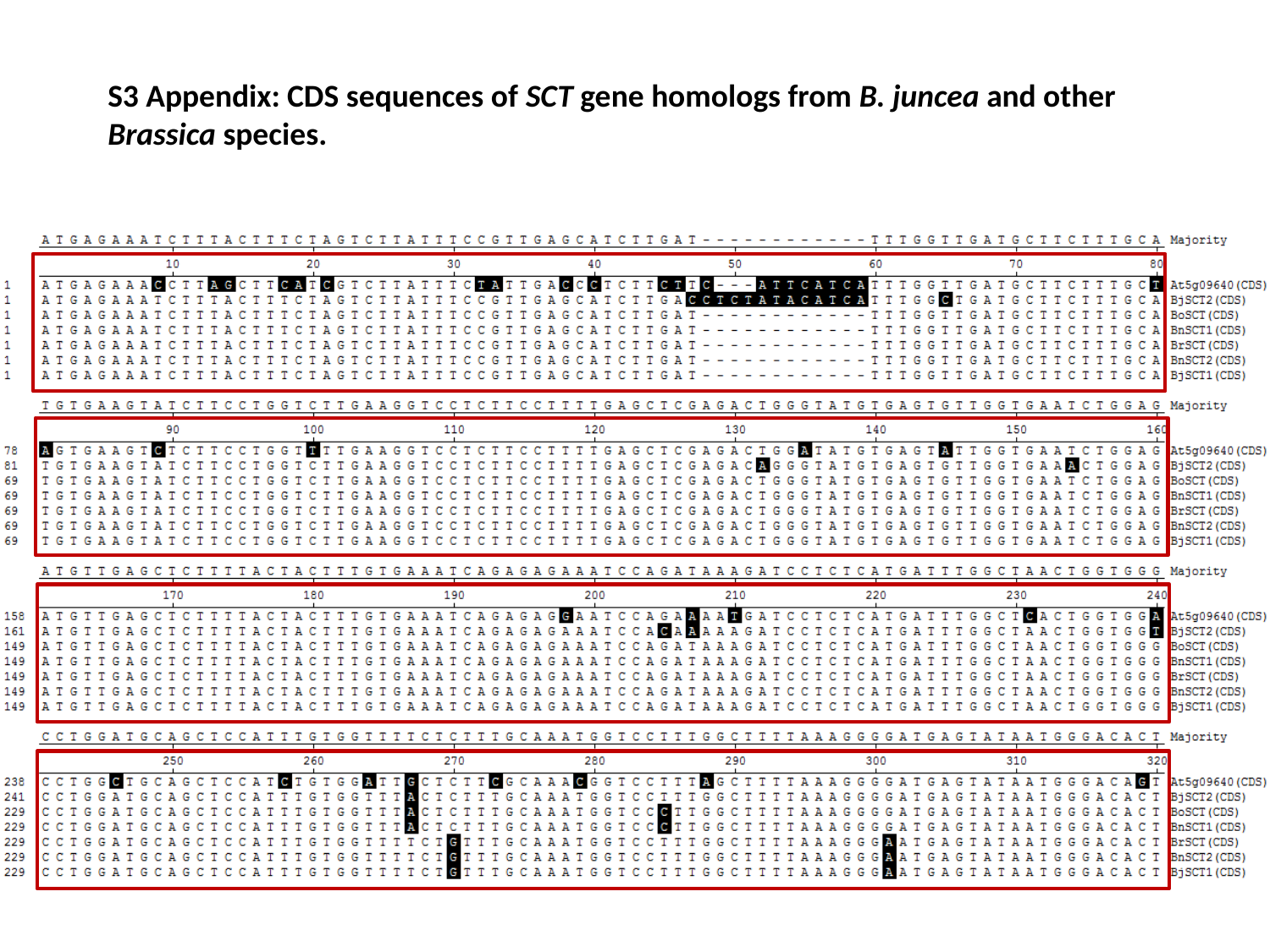

S3 Appendix: CDS sequences of SCT gene homologs from B. juncea and other Brassica species.

## Slide 2
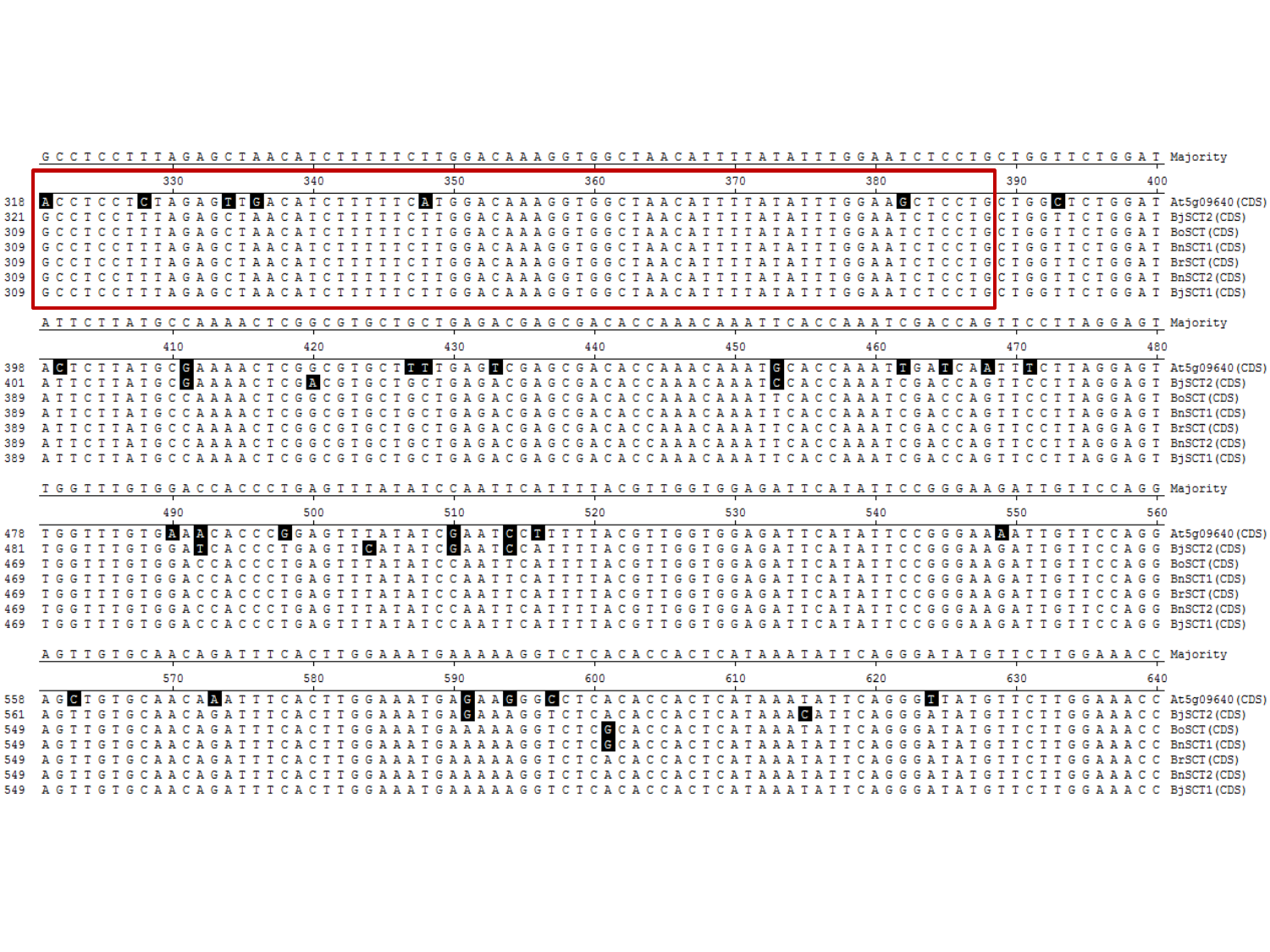

## Slide 3
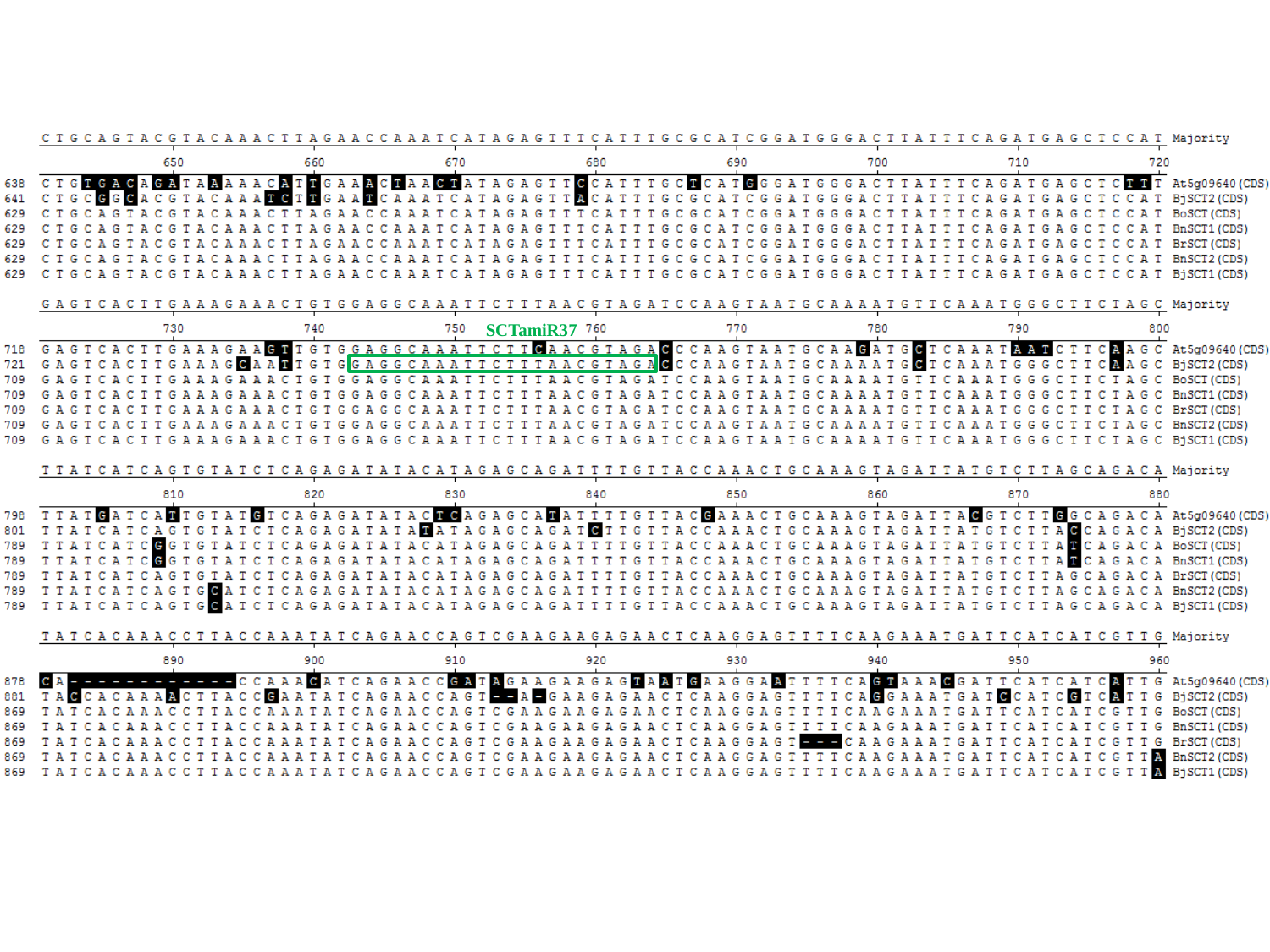

SCTamiR37

## Slide 4
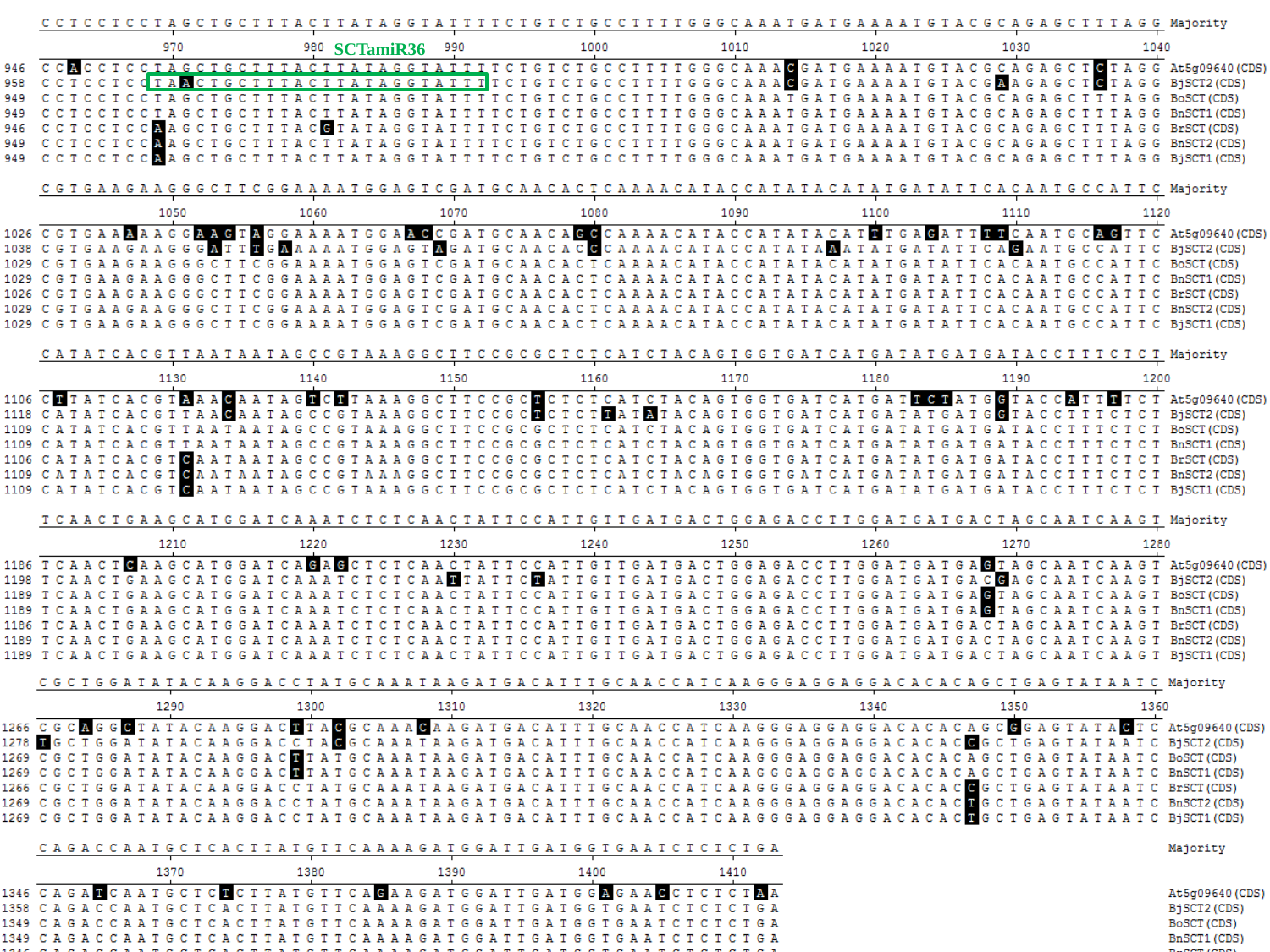

SCTamiR36

## Slide 5
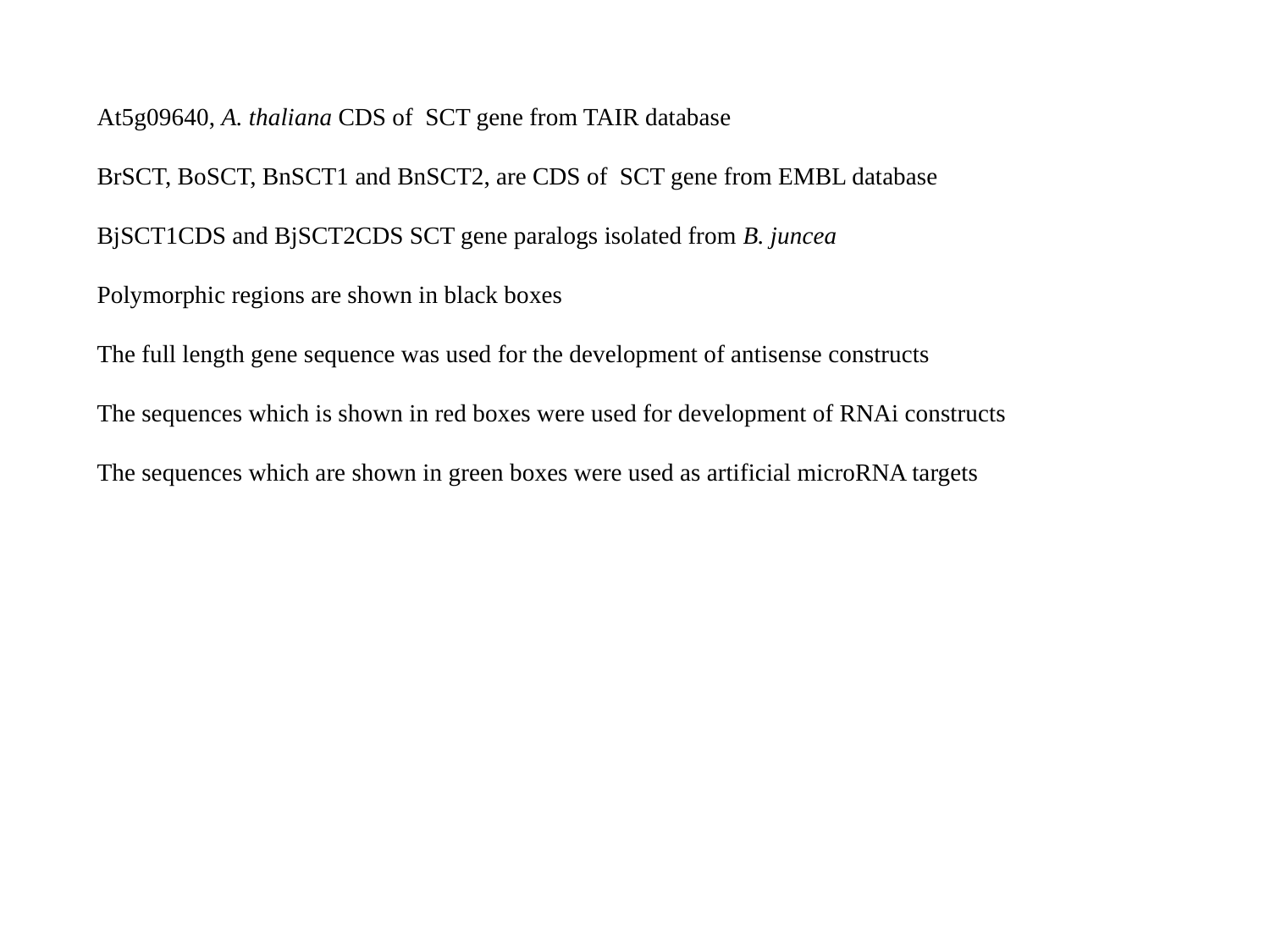

At5g09640, A. thaliana CDS of SCT gene from TAIR database
BrSCT, BoSCT, BnSCT1 and BnSCT2, are CDS of SCT gene from EMBL database
BjSCT1CDS and BjSCT2CDS SCT gene paralogs isolated from B. juncea
Polymorphic regions are shown in black boxes
The full length gene sequence was used for the development of antisense constructs
The sequences which is shown in red boxes were used for development of RNAi constructs
The sequences which are shown in green boxes were used as artificial microRNA targets
